# Health Risk Assessment in Southern Carpathians Small Rural Communities Using Karst Springs as a Drinking Water Source

**DOI:** 10.3390/ijerph19010234

**Published:** 2021-12-26

**Authors:** Ana Moldovan, Anamaria Iulia Török, Ionuț Cornel Mirea, Valer Micle, Oana Teodora Moldovan, Erika Andrea Levei

**Affiliations:** 1 INCDO-INOE 2000, Research Institute for Analytical Instrumentation, 400293 Cluj-Napoca, Romania; ana.moldovan@icia.ro (A.M.); iulia.torok@icia.ro (A.I.T.); 2 Faculty of Materials and Environmental Engineering, Technical University, 400641 Cluj-Napoca, Romania; valer.micle@imadd.utcluj.ro; 3 Department of Geospeleology and Paleontology, Emil Racovita Institute of Speleology, 050711 Bucharest, Romania; ionut.cornel.mirea@gmail.com; 4 Cluj-Napoca Department, Emil Racovita Institute of Speleology, 400006 Cluj-Napoca, Romania; oanamol35@gmail.com

**Keywords:** water quality, health risk assessment, drinking water, karst spring, rural communities

## Abstract

The chemical quality of waters from eight karst springs from the Southern Carpathians and the health risk of small rural communities using these springs as a drinking water source were assessed. The results indicated that the spring waters in the studied area are chemically suitable to be used as drinking water and pose no health risks for adults and children. The spring water can be generally described as having circumneutral pH, Ca-Mg-HCO_3_^−^ facies, excellent to good palatability, and low trace metal and nitrate content. The variation of chemical parameters between spring and autumn was low. These springs could become appropriate drinking water sources for the neighboring rural communities after the assessment of their microbiological status and, if it is the case, proper water treatment. Moreover, periodic monitoring of the water’s chemical parameters, mostly nitrates, as well as the establishment of a protected area near the springs to prevent the negative impact of anthropogenic sources on water quality is recommended.

## 1. Introduction

Access to water with appropriate quality is a worldwide priority, as it is crucial for human health, as well as for economic and social development [1,2,3]. Groundwater is an important source of excellent quality water, albeit vulnerable to both depletion and degradation. The groundwater composition depends on the rock type that hosts the water, the residence time, the original composition of the groundwater, the water flow path, and the type of land use and land use practices in its vicinity [4,5]. Karst areas hold important sources of drinking water all over the world, as they can store large volumes of water [2,6]. Karst aquifers exhibit more complex behavior than other aquifers and are highly vulnerable to pollution, especially by infiltrations of agricultural or sewage runoff [2,3]. The rapid movement of water from the surface to underground and the short water retention time allows for the easy transfer of pollutants and favors the alteration of the water quality [2]. Waters with unknown or inappropriate quality may be used as drinking waters sources by local communities, especially in remote rural areas [7]. Regardless of the socio-economic status, country, or region, communities reliant on drinking water with unknown quality may be susceptible to an increased health risk due to a lack of awareness and false perception of the existing hazards. In Romania, despite the important steps taken in the last decade to ensure access to safe drinking waters, there are still a high number of small rural communities that rely on individual wells, natural sprigs, or improvised local water distribution networks with unknown or poorly monitored quality waters. Such rural communities are often exposed to potential health risks through the consumption of inadequate quality drinking waters. A previous survey of water quality in four karst springs in south-eastern Romania revealed high nitrate concentrations in two of the springs and the presence of potential non-carcinogenic health risks posed by the consumption of these waters without proper pretreatment [4].

This current work investigated the physico-chemical water quality of eight karst springs from the Southern Carpathians and the potential health risk of small rural communities that use these karst springs as a drinking water source. To the best of our knowledge, the water chemistry of this springs was not studied before, the locals being unaware of the water quality and of the potential health risks arising from the use of these springs as a drinking water source.

## 2. Materials and Methods

### 2.1. Geological and Geographical Settings of the Study Area

**Figure 1:**
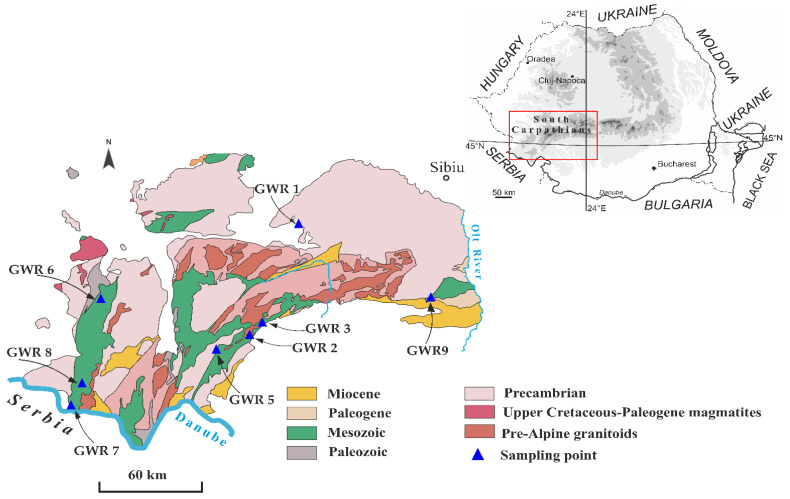
Generalized geological settings of the Southern Carpathians (simplified after Săndulescu et al., 1978 [8]), where the studied springs (GWR1, GWR2, GWR3, GWR5, GWR6, GWR7, GWR8, GWR9) are located.

The studied karst springs are distributed along the southern slope of the Southern Carpathians. This region is inhabited by rural communities where the local springs are often used for drinking and household purposes. The springs were selected according to their use as a drinking water source with easy access. The main types of land cover in the areas where the springs are located are forest, natural grasslands, and non-irrigated arable lands, while the presence of discontinuous industrial zones is low. The main human activities that may affect the spring water quality are agriculture, forest cutting, pasturage, septic systems, and domestic wastes. Most of the karst landscape and landforms on the southern slope of the Southern Carpathians are developed in reef limestones associated with Late Jurassic to Early Cretaceous rocks (Figure 1). 

Some of the studied springs are connected with large caves systems (e.g., Izverna, Fușteica). The Southern Carpathians are known to be one of the most massive and highest mountains of the Romanian Carpathians, being widely influenced by different climatic and hydrological patterns from East to West. From a climate perspective, the studied springs belong to karst areas of different climatic influences, as the Carpathian range acts as a barrier for the atmospheric flow. The hydrology of Southern Carpathians is closely related to the structural and petrographic patterns of the mountain, and the majority of the springs and rivers drain into the Danube. The aquifers (local or discontinuous) that fed the springs are formed in porous formations such as limestones and fissured rocks, usually represented by gravels, sands, limestones, sandstones, and conglomerates. 

### 2.2. Sampling and Analysis

A total of 32 water samples were collected from eight springs (Table 1) in October 2019 (A19), May 2020 (S20), November 2020 (A20), and May 2021 (S21). The GWR1, GWR2, GWR5, GWR6, and GWR8 springs are collected and pumped in local drinking water distribution networks, while the other springs (GWR3, GWR7, and GWR9) flow freely through a concrete basin. The local population use these waters for drinking and household activities. 

All studied springs are perennial, although their discharge may differ between the spring and autumn seasons (Table 1). Based on the median water discharge measured during the sampling campaigns, GWR5 was classified as a 3rd magnitude (28–280 L s^−1^), GWR2 as 4th magnitude (6.3–28 L s^−1^), GWR3 as 5th magnitude (0.63–6.3 L s^−1^), GWR1, GWR6, and GWR8 as 6th magnitude (63–630 mL s^−1^), while GWR7 and GWR9 as 7th magnitude (8–63 mL s^−1^) springs.

The water samples were kept in polyethylene bottles at 4 °C during transportation and analyzed within 24 h. The pH and electrical conductivity (EC) were determined in situ using a PC6 tester kit (Dostmann, Wertheim-Reicholzheim, Germany). Total dissolved solids (TDS) were determined by gravimetry, while bicarbonates (HCO_3_^−^) were determined by titration with 0.1 N HCl in the presence of a bromocresol green indicator. The turbidity (TU) of the spring waters was measured by a Turb 555 IR turbidimeter (WTW, Weilheim, Germany). The Na, Mg, K, and Ca concentrations were measured using an Optima 5300 DV inductively coupled plasma atomic emission spectrometer (ICP-OES, Perkin Elmer, Waltham, MA, USA), while the Al, Fe, Cr, Mn, Ni, Cu, Zn, Sr, Ba, Pb, Cd, and As concentrations were measured using an ELAN DRC II inductively coupled mass spectrometer (ICP-MS, Perkin Elmer, Waltham, MA, USA). 

For the metal determination, the water samples were filtered through 0.45 µm cellulose acetate membrane filters and acidified to pH < 2 with 65% HNO_3_. In order to avoid the polyatomic ^40^Ar^35^Cl^+^ interference on the ^75^As isotope, As was determined as a polyatomic ion (^75^As^16^O)^+^ using the dynamic reaction cell in a DRC mode (RPq = 0.45, O_2_ reaction gas 0.4 mL min^−1^). The anions (Cl^−^, NO_3_^−^, NO_2_^−^, PO_4_^3−^, SO_4_^2−^, F^−^) concentrations were measured using a 761 Compact ion chromatography (Metrohm, Herisau, Switzerland) after filtering the samples through 0.45 µm cellulose acetate membrane filters. Total hardness (TH) was computed as equivalent CaCO_3_, based on Ca and Mg concentrations, according to Clesceri [9]. 

The limit of detection (LOD) was calculated as the ratio between 3 times the standard deviation resulting from 10 measurements of the reagent blank and the slope of the calibration curve (Table 2) [10,11].

To ensure the quality of the results, calibration standards, duplicate samples, and procedural blank measurements were used. Standard solutions containing 1000 mgL^−1^ Cl^−^, 1000 mgL^−1^ NO_3_^−^, 1000 mgL^−1^ PO_4_^3−^, 1000 mgL^−1^ F^−^, and 1000 mgL^−1^ SO_4_^2−^ (Certipur, Merck, Darmstadt, Germany) and nitrite standard solution (1000 mgL^−1^ NO_2_^−^, Certipur, Merck, Darmstadt, Germany) were used for the calibration of the ion chromatograph. Multi-element Calibration Standard 3 (Merck, Darmstadt, Germany) was used for the calibration of the spectrometers. Cal Kit Turb P 555 IR (WTW, Weilheim, Germany) calibration set containing standards of 0.02–1750 nephelometric turbidity units (NTU) were used for the calibration of the turbidimeter.

The accuracy of the anions analysis was checked by analyzing SPS-NUTR WW1 Batch 115 wastewater reference materials (Spectrapure Standards, Oslo, Norway), while, for the metal determination, 1643f NIST freshwater certified reference (National Institute of Standards and Technology, Gaithersburg, MD, US) was used. The mean recovery values ranged between 89–102% for anions and 94–105% for metals. The cation-anion balance showing the percent differences between the sum of cation and anion equivalents fell within ±5% of the acceptable limit, indicating that there was no important analytical error in the measurement of major ions [12]. All of the used reagents were of analytical grade. Ultrapure water from a PureLab system (Veolia Environnement, Paris, France) was used for all dilutions and for the preparation of the standard solutions.

### 2.3. Water Quality Index

Water Quality Index (WQI) is a useful tool for assessing the overall quality of water based on an index number [13,14,15,16]. WQI classifies water into five categories: excellent (0–25), good (26–50), poor (51–75), very poor (76–100), and unsuitable (>100) for human consumption [17]. The WQI was computed according to the following steps: (i) assignment of weights *w_i_* for each parameter based on their importance for water quality; (ii) calculation of the relative weight *W_i_* and the establishment of the quality rating *q_i_* (Equations (1) and (2)); (iii) calculation of the subindex *SI_i_* for each indicator (Equation (3)); (iv) calculation of WQI (Equation (4)) [13,18].

(1) $$W_{i}=\frac{w_{i}}{\sum_{i=1}^{n}w_{i}}$$

(2) $$q_{i}=\frac{C_{i}}{S_{i}}\times 100$$

(3) $$SI_{i}=W_{i}\times q_{i}$$

(4) $$WQI=\sum_{i=1}^{n}SI_{i}$$

where *w_i_* is the weight of each parameter, *W_i_* is the relative weight, *q_i_* is the rating for each parameter, *C_i_* is the measured concentration, and *S_i_* is the guideline value according to the drinking water quality guidelines established by the World Health Organization (WHO) [19] or the parametric values set by Directive 2020/2184 on the quality of water intended for human consumption [20]. *SI_i_* represents the subindex of each parameter (Table 3).

### 2.4. Human Health Risk Assessment

Health risk assessment uses a mathematical model to quantify the risk to human health following exposure to contaminated water [20,21]. Previous studies reported the negative impact on human health of groundwater pollutants through oral ingestion and dermal contact [22]. The non-carcinogenic risks via ingestion and dermal contact were calculated for two age groups: children (0–21 years) and adults (21–72 years). The Hazard Quotient (*HQ*) was calculated for oral and dermal exposure using Equation (5), based on the Average Daily Dose (*ADD*) (Equations (6) and (7)) [18].

(5) $$HQ=\frac{ADD}{R_{f}D}$$

(6) $$ADD_{oral}=\frac{CW*IR*EF*ED}{BW*AT}$$

(7) $$ADD_{dermal}=\frac{CW*SA*K_{p}*ET*EF*ED*10^{-3}}{BW*AT}$$

where *CW* is the concentration of pollutants in the water [µg L^−1^], *IR* is the ingestion rate [L day^−1^], *EF* and *ED* are the exposure frequency [days years^−1^] and duration [years], *BW* is the body weight [kg], *AT* is the average time [days], *SA* is the exposed skin area [cm^2^], *K_p_* is the dermal permeability coefficient in the water [cm h^−1^], and *ET* is the exposure time [h day^−1^] (Table 4) [23]. Potentially toxic elements that were undetected in the analyzed spring waters (As, Pb, Cd) were not included in the health risk assessment.

The overall potential of more than one element for non-carcinogenic effects was calculated by the sum of *HQ* for each pollutant and expressed as the hazard index (*HI*) (Equation (8)). Total Hazard Index (*THI*) was calculated according to Equation (9) [23]. *HQ*, *HI*, or *THI* values above 1.0 indicate the presence of a non-carcinogenic health risk [23].

(8) HI=∑HQ

(9) $$THI=HI_{oral}+HI_{dermal}$$

## 3. Results and Discussion

### 3.1. Hydro-Chemical Typology of the Studied Spring Waters

**Figure 2:**
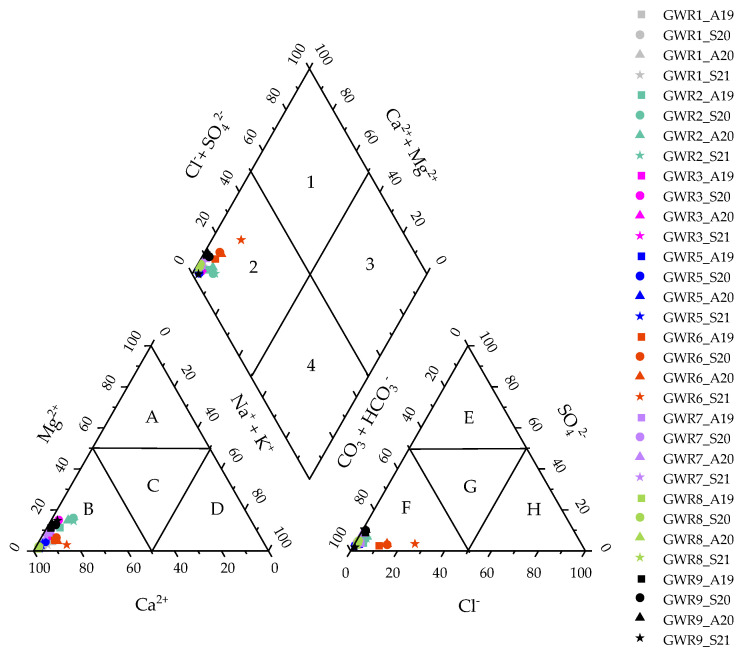
Piper diagram of the studied spring waters as well as the dominant cation and anion fields (A-Magnesium type; B-Calcium type; C-No dominant type; D-Sodium type; E-Sulfate type; F-Bicarbonate type; G-No dominant type; H-Calcium, Magnesium bicarbonate type) and the hydrochemical facies (1-Calcium, magnesium sulfate; 2-Calcium, magnesium bicarbonate; 3-Sodium chloride; 4- Sodium bicarbonate).

The geochemical facies of the studied spring waters were generated by plotting the concentrations of major cations and anions in the Piper trilinear diagram (Figure 2).

This revealed that all of the springs have similar Ca-Mg-HCO_3_^−^ facies, specific for karst areas. Additionally, the dominance of the alkaline-earth metals over the alkali elements (Ca + Mg > Na + K) and of weak acids (HCO_3_^−^) over the strong acids (Cl^−^ + SO_4_^2−^) was noticed, suggesting the predominant influence of rock weathering on the water chemistry. The dominance of Ca and Mg in the spring waters suggested an inverse ion exchange process [28,29], while the high value of HCO_3_^−^ indicated the dissolution of limestone in a karst aquifer [30]. There was no change in the hydrochemical facies between autumn and spring in any of the springs, which indicated that the major ions are of natural origin. 

### 3.2. Physico-Chemical Parameters and Water Quality

**Figure 3:**
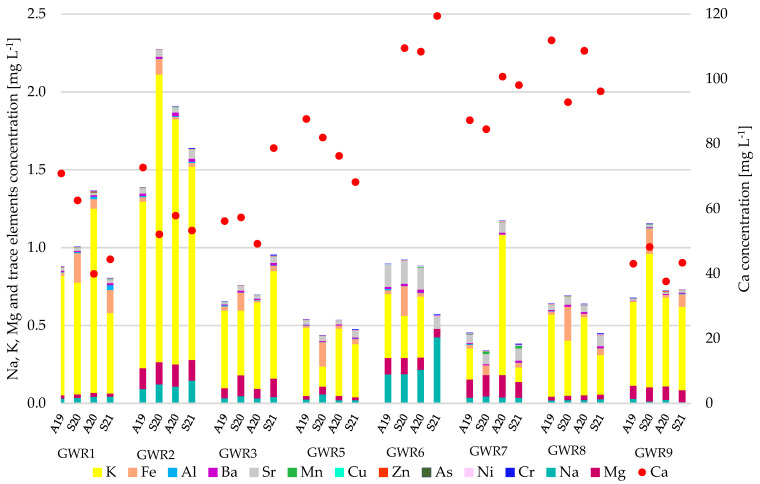
Temporal variation of elements concentration in the studied spring waters.

The physico-chemical parameters of the studied spring waters were compared with the guideline values recommended by the World Health Organization [19] for drinking water and the parametric value for water intended for human consumption set by the European Directive 2020/2184 [20] (Table S1). The waters were circumneutral to slightly alkaline, with pH ranging between 7.2 (GWR6) and 8.3 (GWR1) without important variation between the autumn and spring seasons. The total dissolved solids (TDS) ranged between 120 (GWR3) and 470 (GWR6, GWR8), indicating the concentration of inorganic salts dissolved in water. The palatability of the spring waters based on TDS value ranged from excellent (<300 mgL^−1^) to good (300–600 mg L^−1^). With some exceptions, the highest TDS values were found in Autumn 2019, without a clear seasonal trend. The TU was generally low, ranging from 0.02 to 5.70 NTU. The highest TU values were measured in GWR1. The main chemical components in the spring waters were HCO_3_^−^ (134–372 mg L^−1^) and Ca (37.6–121 mg L^−1^). The highest HCO_3_^−^ and Ca concentrations were recorded in GWR6 and the lowest in GWR9, confirming, as expected, that calcium carbonate is the main constituent of the water in karst areas. The concentrations of Na, Mg, and K were much lower than of Ca in all of the springs (Figure 3). 

Total hardness (TH) in waters reflects the natural dissolution of metal ions, especially Ca and Mg from the rocks that host the water [4]. Both Ca and Mg are essential elements for human health, and there is no evidence of negative health effects caused by water hardness [4,5]. However, the optimum TH value in drinking water is considered 100 mgL^−1^, and a maximum TH should not exceed 300 mgL^−1^ [4,5]. The TH in the spring waters ranged between 105–323 mgL^−1^, values above 300 mgL^−1^ being measured only in GWR6. Based on TH, water classifies as soft (TH < 75), moderately hard (75–150), hard (151–300), or very hard (>300) [5]. Based on the average TH values, GWR1 and GWR 9 were classified as moderately hard, GWR2, GWR3, GWR5, GWR7, and GWR8 were classified as hard waters, and GWR6 was classified as very hard water.

The concentrations of K, Mg, and SO_4_^2−^ were comparable in all of the springs, while those of Na and Cl^−^ were comparable in all of the springs except for GWR6. In the case of GWR6, much higher Na (8.89–20.4 mgL^−1^) and Cl^−^ (25.2–66.0 mgL^−1^) concentrations were observed compared to the other springs (0.38–7.03 mgL^−1^ Na and 0.57–4.65 mgL^−1^ Cl^−^). The concentration of NO_3_^−^ ranged between 0.26 and 11.0 mgL^−1^, the highest values being found in GWR9. The higher nitrate concentrations in some of the spring waters and some of the seasons (e.g., GWR7 in A20) were probably due to the presence of bats in the Gaura cu Muscă Cave, just above the spring [31]. The concentrations of NO_2_^−^ and PO_4_^3−^ were undetectable in all of the studied springs, in all seasons. Sr had the highest concentration in GWR6 in all four seasons and the lowest in GWR9, while Al was the highest in GWR1 (S21) and the lowest in GWR7 (S20). Ba concentration was higher in GWR2 and GWR6 than in the other springs. The Fe concentration varied widely between springs and also between seasons (3.30–219 μgL^−1^), high concentrations being measured in the spring season of 2020 in GWR1, GWR6, GWR7, and GWR8. The concentration of Mn ranged between 0.10 and 4.12 μgL^−1^, except for GWR 7, where it was 3-fold higher. The concentration of Cu, Zn, Ni, and Cr had very small variations between springs, ranging between 0.25–1.38 μgL^−1^ (Cu), 0.60–7.88 μgL^−1^ (Zn), 1.18–7.82 μgL^−1^ (Ni), and 0.200–6.35 μgL^−1^ (Cr), respectively. In areas with limited industrial activities, trace elements can be present in groundwater due to soil infiltration and their migration through meteoric water flow. The concentrations of As, Cd, and Pb were undetectable in all of the spring waters, in all seasons. Generally, the seasonal variations were low for both the major and trace elements. In all the spring waters, the studied parameters were below the guideline value set by the WHO [19] and met the minimum requirements regarding the chemical parameters for water intended for human consumption set by the European Directive 2020/2184 [20].

A previous study on Romanian karst springs used as a drinking water source revealed that the concentrations of radon and radium were below the radioprotection standards recommended by national and European legislation (100 Bq L^−1^); additionally, the radon concentration exceeds the safety limit (11.1 Bq L^−1^) set by the United States Environmental Protection Agency (USEPA) in 31% of the samples in at least one season and two karst springs located in north-western Romania across the four seasons [32].

The WQI (Table 5) showed excellent quality for the studied spring waters and their chemical suitability as drinking water.

The water quality of the studied springs was comparable between the autumn and spring seasons. Although the guideline values set for Mn, Cr, Ni, and Pb by the WHO and EU Directive 2020/2184 are different, the WQI values calculated based on the two thresholds, as well as the water quality status, were similar. The results are similar to the studies on karst springs quality conducted in other regions of Romania (Dobrogea in south-eastern Romania and the Apuseni Mountains in north-western Romania), revealing the water-rock interaction and anthropogenic activities to be the main drivers of water quality [4,31]. In these regions, the quality of the spring waters was excellent and good, with a slight variation of the WQI values [4,31,33].

Generally, the springs with low discharge are more susceptible to human impact than the springs with high discharge, as the low discharge rate may lead to the longer residence of pollutants in the karst network. Conversely, a high discharge rate shortens the contact time between water and host rocks and reduces the probability of major and trace elements as well as pollutants leaching in the water [34]. Based on the quality status of the studied springs, there was no indication of the influence of the discharge rate on the water quality status. GWR5 (3rd magnitude in flow rate) presented the same quality status as GWR7 (7th magnitude in flow rate). Comparing the discharge rates between the sampling seasons (Table 1), GWR5 presented the highest variability. Even so, the discharge rate did not influence the quality status of the spring waters. Considering that the main types of land use in all springs are similar (pastures, forest, natural grasslands, and arable lands), the anthropogenic impact is low and results from agriculture, forest cutting, pasturage, septic systems, and domestic wastes.

**Figure 4:**
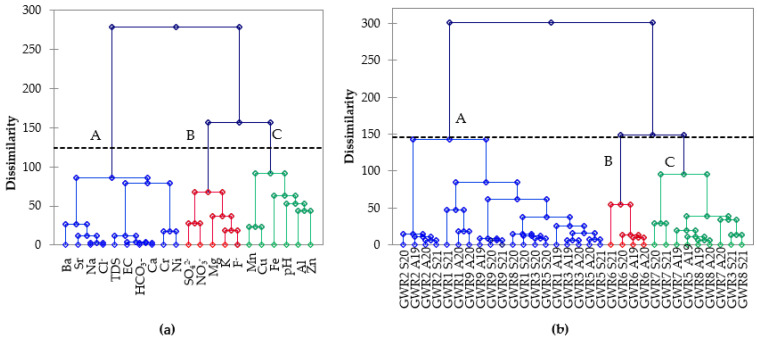
Hierarchical clustering of physico-chemical parameters in spring waters (**a**) and the studied spring samples (**b**).

The hierarchical cluster analysis (HCA) using the Ward method and squared Euclidian distance for linkage was used to group the chemical parameters and the spring waters based on their similarities. In Figure 4a, the influence of three distinct sources on the spring water composition were identified. Cluster A (TDS, EC, HCO_3_^−^, Ca, Ba, Sr, Na, Ni, Cr, and Cl^−^) grouped the parameters that result from water–rock interactions. The main sources of Ca in the spring waters are the dissolution of carbonate minerals (calcite, dolomite, aragonite, vaterite), gypsum, and silicates (anorthite, pyroxene), while HCO_3_^−^ originate from the dissolution of calcite and dolomite [4]. Barium, Sr, and Ni may also originate from the dissolution of carbonate rocks [11]. The dissolution of silicate minerals is enhanced in the presence of Ba in water and further leads to the release of associated trace elements [35]. Sodium results from plagioclases, clays, and feldspars dissolution as well as from cation exchange by clay minerals, while Cl^−^ is derived from rainfall, the dissolution of chloride bearing minerals, and sedimentary rocks [4,36,37]. Cluster B (SO_4_^2−^, NO_3_^−^, Mg, K, and F^−^) grouped the elements that resulted both from geogenic (weathering and dissolution of minerals) and anthropogenic sources (domestic wastes, sewage systems, irrigation-return-flow, and chemical fertilizers). The natural sources of magnesium are dolomite and magmatic minerals such as biotite, hornblende, and olivine, while the anthropogenic ones are fertilizers (magnesium sulphate, sulphate of potash magnesia, magnesium nitrate) or de-icing and anti-clumping agents [4,36,37]. The main potassium sources are feldspars and clays, but also fertilizer (potassium nitrate, sodium nitrate, potassium sulphate) and domestic effluents [4]. Nitrate and sulfate have most likely anthropogenic origins, resulting from agricultural fertilizers (urea, ammonium sulphate), livestock wastes, and sewage discharges. Sulphate may result also by the dissolution of gypsum, anhydrite, and sulfide minerals. [4,36,37]. Cluster C grouped the pH and elements (Fe, Al, Mn, Cu, Zn) that may be leached at low pH or precipitated at high pH and are probably of anthropogenic origin.

Figure 4b classified the springs into three clusters: cluster A groups most of the springs in the majority of the seasons, while the remaining samples were grouped in two other clusters. Cluster B separates GWR6, characterized by high carbonate and high Ca concentrations, in all four of the monitored seasons. This spring has low discharge (6th order of magnitude in flow rate) but is used by the highest number of people. It has the lowest pH (7.2–7.6) and the highest TDS (387–470 mg L^−1^), TH (285–323 mg L^−1^), Na (8.89–20.4 mg L^−1^), Cl^−^ (25.2–66.0 mgL^−1^), and Sr (141–164 mgL^−1^) concentrations. Cluster C groups samples GWR3, GWR5, GWR7, and GWR8, characterized by high carbonate, sulphate, and calcium concentrations in all seasons.

### 3.3. Human Health Risk

**Figure 5:**
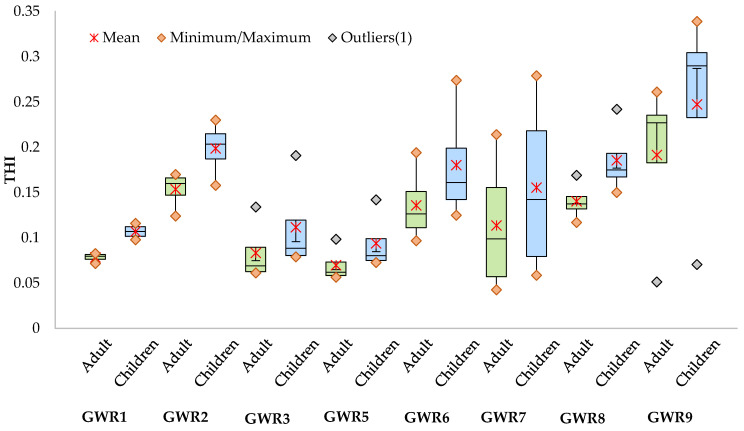
Seasonal variation of total hazard index (THI) for adults and children through dermal and oral exposure to uncontrolled quality spring waters in the studied area.

The ADD values were slightly lower for adults (Table S2) than for children (Table S3), both for oral and dermal exposures. The ADD values for the two age groups for oral exposure were higher than for dermal exposure. The ADD values for oral exposure ranged from 3.14 × 10^−3^ to 3.46 × 10^2^ for adults and from 4.00 × 10^−3^ to 4.40 × 10^2^ for children, while those for dermal exposure ranged from 1.43 × 10^−5^ to 1.63 × 10 for adults and from 2.53 × 10^−5^ to 1.67 × 10 for children. The highest ADD values were obtained for NO_3_^−^, for both adults and children and for both oral and dermal exposures. In the case of metals, the highest ADD values were obtained for Fe and the values were 1–2 orders of magnitude lower for the other elements for both types of exposure and age groups. The values of ADDs varied from site to site and between seasons, without a clear variation pattern. HQ (Tables S4 and S5) and HI (Table S6) values were below the unity threshold for both the oral and dermal exposures and for both adults and children, indicating that neither the metals nor the NO_3_^−^ concentration in the spring waters pose important health risks for the population using these waters. As expected, for each parameter, HQ_oral_ was higher than HQ_dermal_. The highest HQ_oral_ was found for NO_3_^−^ (2.16 × 10^−1^ for adults and 2.75 × 10^−1^ for children) in GWR9, while the highest HQ_dermal_ was found for Cr (1.21 × 10^−2^ for adults and 4.29 × 10^−2^ for children) in GWR6. The HQ_oral_ and HQ_dermal_ were slightly higher for children than for adults, suggesting a higher risk for children than for adults and by ingestion than by dermal exposure. This fact suggests that children are more vulnerable to toxic compounds by ingestion, as they consume more water volume per unit of body weight than adults [38,39]. Qui and Gui [40] also reported that younger people were more susceptible to non-carcinogenic risk than adults in the case of exposure to metals through oral and dermal pathways in Anhui Provence, China. The HI_oral_ was systematically higher than HI_dermal_ for both adults and children. The highest HI_oral_ was found in A19 in GWR9, while the highest HI_dermal_ was in S21 in GWR6 both for adults and children. With some exceptions, the seasonal variation of the HQ and HI were low. Other studies reported groundwater exposure via the oral route as the main factor determining non-carcinogenic risk [41,42]. The THI values varied from 4.26 × 10^−2^ to 2.61 × 10^−1^ for adults and from 5.86 × 10^−2^ to 3.39 × 10^−1^ for children. The seasonal variation of THI was higher for GWR7 and GWR9 than for the other springs (Figure 5).

The highest THI values were found in GWR9 (Figure 5) both for children (3.39 × 10^−1^) and adults (2.61 × 10^−1^), while the lowest THI values were measured in GWR7 (4.26 × 10^−2^ for adults and 5.86 × 10^−2^ for children), indicating that the regular use of these two spring waters poses a probable non-carcinogenic health risk without exceeding the safety threshold. A similar study conducted in Dobrogea, another important karst area from Romania, indicated possible non-carcinogenic risks for both adults and children through oral exposure and the dermal pathway due to elevated nitrates and Cr concentration, respectively [4].

The health risk assessment data based on water chemistry indicated that the studied springs could be used by the local communities as drinking water supply but only after the microbiological quality assessment as well as water treatment and disinfection [43,44]. The regular water quality monitoring could be a preventive measure that will identify any possible contamination. The results of this study could be included in the World Karst Spring (WoKaS) hydrograph database, which collects information on more than 400 springs all over the world [45]. It can also be used for future monitoring and management programs on springs used by local communities as drinking water sources.

## 4. Conclusions

Springs are important sources of water for drinking and domestic usage in the rural communities from the Southern Carpathians. The drinking water sources of several karst springs were analyzed during spring and autumn to investigate their chemical quality and potential health risk for adults and children. The concentrations of the physico-chemical parameters of the spring waters met the guidelines set by the World Health Organization and the parametric values set by the EU Directive 2000/2184. The concentrations of major ions in waters were found to be controlled by water-rock interaction, and no differences in the Ca-Mg-HCO_3_^−^ type water facies were observed between the autumn and spring seasons. The chemical quality of the spring waters used as drinking water was confirmed also by the water quality index, which showed excellent quality status. No important seasonal variation of any of the chemical parameters was observed. According to the hierarchical cluster analysis, the TDS, EC, HCO_3_^−^, Ca, Ba, Sr, Na, Cr, Ni, and Cl^−^ originated from water-rock interactions, the SO_4_^2−^, NO_3_^−^, Mg, K, and F^−^ from both geogenic and anthropogenic sources, and the Fe, Al, Mn, Cu, and Zn from anthropogenic sources. Even from chemical point of view, the use of the spring waters does not pose health risks for the population; their monitoring is a prerequisite for human health. This study shows that water quality assessments, based on WQI and total hazard index, are a useful tool to characterize the springs used as drinking water sources. The obtained results may be the basis for awareness campaigns among the local population that use these springs without knowing its suitability as drinking water.

## Figures and Tables

**Table 1:** Characteristics of the studied springs.

Spring	Site	Locality	Spring Type	Geographical Coordinates	Altitude (m.a.s.l.)	Discharge (L min^−1^)				Main Type of Land Use	Approx. No. of People Using the Water
						A19	S20	A20	S21		
GWR1	Șura Mare Cave	Ohaba Ponor	Improvised plastic tube from the cave	45°31′35.29′′ N 23°8′26.60′′ E	436	20	15	25	20	Forest/Pastures/Agriculture	289
GWR2	Fușteica Cave	Izvarna	Concrete basin with a metal tube	45°1′46.88′′ N 22°54′9.16′′ E	214	480	600	1400	600	Forest/Natural grasslands	338
GWR3	Tismana	Tismana	Concrete basin with a metal tube	45°4′46.59′′ N 2°55′44.02′′ E	276	50	60	50	120	Forest/Natural grasslands	1745
GWR5	Izverna Cave	Isverna	Natural karstic outlet	44°58′49.15′′ N 22°37′7.46′′ E	465	3600	7500	1800	3000	Forest/Pastures	599
GWR6	“Sfânta Maria” Carasova	Carașova	Metal tube	45°11′16.66′′ N 21°51′16.07′′ E	220	25	36	30	20	Forest/Pastures/Agriculture	2341
GWR7	Gaura cu Muscă Cave	No locality, touristic cave	Concrete basin with a metal tube	44°39′52.46′′ N 21°41′56.15′′ E	90	0.5	0.8	0.2	4.0	Forest/ Agriculture/ Pastures	360
GWR8	Padina Matei Cave	Padina Matei	Concrete basin with a metal tube	44°45′43.55′′ N 21°44′28.02′′ E	578	30	90	30	40	Forest/Pastures/Woodland-shrubs	951
GWR9	Bistriței Gorges	No locality, touristic place	Concrete basin with a metal tube	45°11′59.60′′ N 24°1′49.81′′ E	650	0.2	1.3	0.2	3.0	Forest/ Fruit tree plantation	926

**Table 2:** The limit of detection (LOD) of the studied parameters in spring water.

Parameter	LOD	Parameter	LOD
HCO_3_^−^ (mg L^−1^)	20	Fe (μg L^−1^)	0.10
TU (NTU)	0.01	Al (μg L^−1^)	2.00
Na (mg L^−1^)	0.01	Cr (μg L^−1^)	0.19
Mg (mg L^−1^)	0.009	Mn (μg L^−1^)	0.08
K (mg L^−1^)	0.012	Ni (μg L^−1^)	0.13
Ca (mg L^−1^)	0.004	Cu (μg L^−1^)	0.21
TDS (mg L^−1^)	3.0	Zn (μg L^−1^)	0.31
Cl^−^ (mg L^−1^)	0.02	Sr (μg L^−1^)	0.10
NO_3_^−^ (mg L^−1^)	0.01	Ba (μg L^−1^)	0.16
SO_4_^2−^ (mg L^−1^)	0.03	Pb (μg L^−1^)	0.11
F^−^ (mg L^−1^)	0.01	As (μg L^−1^)	0.27
NO_2_^−^ (mg L^−1^)	0.05	Cd (μg L^−1^)	0.07
PO_4_^3−^ (mg L^−1^)	0.08		

**Table 3:** Chemical parameters, weight (*w_i_*), relative weights (*W_i_*), and guideline value (*S_i_*) used for the calculation of Water Quality Index.

Parameter	Units	*w_i_*	*W_i_*	*S_i_* *	*S_i_* **
pH	-	5	0.09	6.5–8.5	6.5–9.5
TDS	mg L^−1^	5	0.09	1000	-
Ca	mg L^−1^	3	0.05	75	-
Mg	mg L^−1^	2	0.04	30	-
Na	mg L^−1^	2	0.04	200	200
K	mg L^−1^	2	0.04	12	-
Cl^−^	mg L^−1^	4	0.07	250	250
SO_4_^2−^	mg L^−1^	1	0.02	250	250
NO_3_^−^	mg L^−1^	4	0.07	50	50
Ni	µg L^−1^	4	0.07	70	20
As	µg L^−1^	4	0.07	10	10
Fe	µg L^−1^	4	0.07	200	200
Pb	µg L^−1^	4	0.07	10	5
Mn	µg L^−1^	4	0.07	100	50
Cu	µg L^−1^	4	0.07	2000	2000
Cr	µg L^−1^	4	0.07	50	25
		∑wi=56	∑Wi=1		

* *S_i_* guideline value according to the World Health Organizations Guidelines for Drinking-Water Quality [19]. ** *S_i_* parametric value according to European Directive 2020/2184 [20].

**Table 4:** Input variables used to calculate the Hazard Quotient for adults and children.

Parameters		Units	Values				References
			Adult		Children		
Ingestion rate (*IR*)		L day^−1^	2.2		1		[24,25]
Exposure frequency (*EF*)		days year^−1^	Oral	Dermal	Oral	Dermal	[21,24]
			365	350	365	350	
Exposure duration (*ED*)		year	Oral	Dermal	Oral	Dermal	[21,24]
			70	30	10	6	
Surface skin (*SA*)		cm^2^	18,000		6600		[23]
Exposure time oral (*ET*)		h day^−1^	0.58		1		[23]
Dermal permeability coefficient in water (*K_p_*)	Al	cm h^−1^	0.001		0.001		[23,26]
	Ba		0.001		0.001		
	Mn		0.001		0.001		
	Fe		0.001		0.001		
	Cu		0.001		0.001		
	Zn		0.0006		0.0006		
	Ni		0.0002		0.0002		
	Cr		0.002		0.002		
	NO_3_^─^		0.006		0.006		
Body weight (*BW*)		kg	70		25		[21]
Average time (*AT*)		days	Oral	Dermal	Oral	Dermal	[21]
			25,550	10,950	3650	2190	
Reference dose (*RfD*)		μg kg^−1^ day^−1^	Oral	Dermal	Oral	Dermal	[23,26,27]
	Al		1000	200	1000	200	
	Ba		200	14	200	14	
	Mn		24	0.96	24	0.96	
	Fe		700	140	700	140	
	Cu		40	8	40	8	
	Zn		300	60	300	60	
	Ni		20	0.8	20	0.8	
	Cr		3	0.075	3	0.075	
	NO_3_^─^		1600	1600	1600	1600	

**Table 5:** Water Quality Index (WQI) and quality status (QS) of the studied karst spring waters calculated using WHO guidelines [19] and Directive 2020/2184 parametric values [20].

Spring	WQI *				WQI **				QS *	QS **
	A19	S20	A20	S21	A19	S20	A20	S21		
GWR1	15.0	14.6	12.3	14.6	15.0	14.4	12.2	14.6	Excellent	Excellent
GWR2	18.0	16.9	17.3	16.2	18.0	16.4	17.2	16.7	Excellent	Excellent
GWR3	13.4	13.9	13.2	15.6	12.9	13.4	12.8	15.9	Excellent	Excellent
GWR5	18.5	14.0	14.6	14.2	21.3	13.8	14.7	15.1	Excellent	Excellent
GWR6	18.9	18.6	18.6	20.7	19.3	18.8	19.3	21.6	Excellent	Excellent
GWR7	17.2	16.4	19.1	20.1	15.2	17.7	19.7	22.7	Excellent	Excellent
GWR8	19.0	17.3	18.0	18.0	19.4	17.1	18.4	19.9	Excellent	Excellent
GWR9	19.2	18.3	18.4	16.3	19.6	18.3	18.3	16.3	Excellent	Excellent

* calculated based on guideline value according to World Health Organizations Guidelines for Drinking-Water Quality [19]. ** calculated based on parametric value according to European Directive 2020/2184 [20].

## Data Availability

The data presented in this study are available on request from the corresponding author.

## Supplementary Materials

The following are available online at https://www.mdpi.com/article/10.3390/ijerph19010234/s1, Table S1: Physico-chemical parameters of spring waters collected in October 2019 (A19), May 2020 (S20), November 2020 (A20), and Spring 2021 (S21), Table S2: The average daily dosage (ADD) of metals and nitrate through oral and dermal pathways for those in the adult age category exposed to spring waters collected in October 2019 (A19), May 2020 (S20), November 2020 (A20), and Spring 2021 (S21), Table S3: The average daily dosage (ADD) of metals and nitrate through the oral and dermal pathways for those in the children age category exposed to spring waters collected in October 2019 (A19), May 2020 (S20), November 2020 (A20), and Spring 2021 (S21), Table S4: The hazard quotient (HQ) values of metals and nitrate through the oral and dermal pathways for those in the adult age category exposed to spring waters collected in October 2019 (A19), May 2020 (S20), November 2020 (A20), and Spring 2021 (S21), Table S5: The hazard quotient (HQ) of metals and nitrate through the oral and dermal pathways for those in the children age category exposed to spring waters collected in October 2019 (A19), May 2020 (S20), November 2020 (A20), and Spring 2021 (S21), Table S6: The hazard index (HI) for the oral and dermal pathways and the total hazard index (THI) for those in the adult and children age categories exposed to spring waters collected in October 2019 (A19), May 2020 (S20), November 2020 (A20), and Spring 2021 (S21).
