# Supplementary material for: Health Risk Assessment in Southern Carpathians Small Rural Communities Using Karst Springs as a Drinking Water Source
Source: Int J Environ Res Public Health. 2021 Dec 26;19(1):234. doi: 10.3390/ijerph19010234 (PMC8744857; doi:10.3390/ijerph19010234)
Supplement: Supplementary file 1 [file ijerph-19-00234-s001.zip › ijerph-1464539-supplementary.pdf]

Table S1. Physico-chemical parameters of spring waters collected in October 2019 (A19), May 2020 (S20), November 2020 (A20), and Spring 2021 (S21).

Spring	Season	pH	EC	HCO_3^-	TU	TH	Na	Mg	K	Ca	Fe	Al	Cr	Mn	Ni	Cu	Zn	Sr	Ba	Pb	As	Cd	TDS	Cl^-	SO_4^{2-}	NO_3^-	NO_2^-	PO_4^{3-}	F^-
			μS cm⁻¹	mgL⁻¹	NTU	mgL⁻¹	mgL⁻¹	mgL⁻¹	mgL⁻¹	mgL⁻¹	μgL⁻¹	μgL⁻¹	μgL⁻¹	μgL⁻¹	μgL⁻¹	μgL⁻¹	μgL⁻¹	μgL⁻¹	μgL⁻¹	μgL⁻¹	μgL⁻¹	μgL⁻¹	mgL⁻¹	mgL⁻¹	mgL⁻¹	mgL⁻¹	mgL⁻¹	mgL⁻¹	mgL⁻¹
**GWR1**	**A19**	8.30	326	220	3.60	181	1.44	0.95	0.82	70.9	20.2	4.46	1.11	0.60	2.70	1.05	2.00	18.5	7.50	<LOD	<LOD	<LOD	340	1.07	6.55	2.70	<LOD	<LOD	0.07
	**S20**	8.10	317	210	3.54	161	1.64	1.07	0.78	62.6	190	7.25	1.59	0.84	1.55	0.42	1.50	22.5	6.70	<LOD	<LOD	<LOD	205	0.95	7.10	1.83	<LOD	<LOD	0.06
	**A20**	8.00	211	134	2.18	105	2.01	1.16	1.25	39.9	60.9	16.1	2.59	1.71	1.29	0.57	7.88	14.2	10.5	<LOD	<LOD	<LOD	150	1.13	6.30	1.93	<LOD	<LOD	0.12
	**S21**	8.30	227	140	5.70	115	2.09	0.88	0.58	44.4	148	31.9	1.72	2.10	2.48	0.38	1.90	24.2	12.8	<LOD	<LOD	<LOD	160	0.93	6.99	2.06	<LOD	<LOD	0.13
**GWR2**	**A19**	7.50	440	268	0.20	208	4.37	6.39	1.30	72.7	28.8	8.56	0.20	0.50	2.99	0.35	0.80	36.4	14.7	<LOD	<LOD	<LOD	377	4.39	10.1	5.44	<LOD	<LOD	0.25
	**S20**	7.90	349	212	0.82	158	5.87	6.77	2.11	52.1	101	0.92	0.30	0.38	1.25	0.25	1.10	44.3	12.1	<LOD	<LOD	<LOD	220	3.92	10.8	6.88	<LOD	<LOD	0.18
	**A20**	7.80	382	207	0.40	173	5.15	6.77	1.82	57.9	16.2	5.70	0.39	1.82	2.66	0.49	0.68	34.0	23.1	<LOD	<LOD	<LOD	230	4.35	12.5	7.20	<LOD	<LOD	0.24
	**S21**	7.70	366	207	0.32	159	7.03	6.24	1.52	53.3	23.9	8.90	3.49	0.40	3.31	0.40	1.50	58.5	18.5	<LOD	<LOD	<LOD	230	4.65	11.0	5.63	<LOD	<LOD	0.21
**GWR3**	**A19**	7.80	246	183	0.06	153	1.61	2.97	0.60	56.2	26.9	7.94	0.30	0.40	1.18	0.30	0.70	15.0	5.30	<LOD	<LOD	<LOD	197	1.20	5.97	2.55	<LOD	<LOD	0.10
	**S20**	7.70	359	222	0.10	170	2.20	6.41	0.60	57.3	115	1.18	0.22	0.30	1.51	0.28	0.80	30.8	12.0	<LOD	<LOD	<LOD	205	0.92	6.50	3.00	<LOD	<LOD	0.09
	**A20**	8.20	226	174	0.11	135	1.50	2.91	0.65	49.2	13.3	3.50	0.26	1.31	1.63	0.35	0.60	22.6	7.52	<LOD	<LOD	<LOD	120	0.89	6.10	2.58	<LOD	<LOD	0.17
	**S21**	7.90	453	293	0.12	220	1.93	5.59	0.85	78.7	34.2	1.50	5.04	0.30	4.87	0.32	1.60	40.7	16.3	<LOD	<LOD	<LOD	300	0.93	7.54	2.83	<LOD	<LOD	0.12
**GWR5**	**A19**	7.80	391	250	0.02	223	1.20	1.00	0.49	87.7	13.8	2.04	0.20	0.30	3.26	0.28	0.90	29.2	5.30	<LOD	<LOD	<LOD	439	2.09	8.28	2.39	<LOD	<LOD	0.06
	**S20**	7.60	395	262	0.40	215	2.73	2.39	0.24	82.0	157	0.74	0.28	0.36	2.27	0.36	1.00	32.1	6.37	<LOD	<LOD	<LOD	245	0.92	5.45	1.95	<LOD	<LOD	0.05
	**A20**	8.20	412	244	0.05	196	1.00	1.20	0.48	76.3	19.3	0.80	0.57	1.45	3.24	0.25	0.80	22.0	7.21	<LOD	<LOD	<LOD	220	1.70	6.90	2.34	<LOD	<LOD	0.11
	**S21**	7.80	359	212	0.46	174	0.94	0.89	0.38	68.2	32.6	3.10	3.81	0.20	5.02	0.34	1.10	41.9	7.50	<LOD	<LOD	<LOD	225	1.14	6.92	1.93	<LOD	<LOD	0.11
**GWR6**	**A19**	7.20	608	372	0.16	323	8.89	5.00	0.70	121	24.3	7.34	0.30	0.45	4.49	0.34	0.90	141	13.6	<LOD	<LOD	<LOD	387	25.2	10.7	5.89	<LOD	<LOD	0.12
	**S20**	7.60	628	336	0.03	295	9.01	4.92	0.56	110	191	1.79	0.20	0.32	2.43	0.46	0.70	149	14.4	<LOD	<LOD	<LOD	390	33.0	9.59	4.76	<LOD	<LOD	0.09
	**A20**	7.60	633	342	0.11	285	10.4	3.67	0.69	108	22.6	2.70	0.48	4.12	4.68	0.64	0.96	141	21.2	<LOD	<LOD	<LOD	390	33.0	11.0	3.50	<LOD	<LOD	0.14
	**S21**	7.40	694	313	0.72	308	20.4	2.55	0.33	119	32.3	3.90	6.35	0.20	7.82	0.50	1.00	164	25.1	<LOD	<LOD	<LOD	470	66.0	12.0	4.70	<LOD	<LOD	0.06
**GWR7**	**A19**	7.40	525	290	1.91	241	1.71	5.60	0.35	87.3	22.8	6.07	1.00	2.20	4.11	0.52	0.80	57.0	4.50	<LOD	<LOD	<LOD	417	3.03	10.7	0.87	<LOD	<LOD	0.10
	**S20**	7.70	517	319	0.40	238	2.17	6.50	0.06	84.5	187	0.98	1.50	15.5	2.70	1.38	1.10	65.2	6.81	<LOD	<LOD	<LOD	305	1.73	12.7	0.26	<LOD	<LOD	0.08
	**A20**	8.00	482	305	0.10	281	1.83	6.84	1.08	101	4.83	0.49	1.89	1.67	4.46	0.43	2.25	66.1	11.4	<LOD	<LOD	<LOD	320	1.43	21.9	8.69	<LOD	<LOD	0.13
	**S21**	7.60	508	324	0.38	265	1.72	4.81	0.23	98.1	30.4	0.90	5.49	16.6	6.41	0.62	1.40	78.8	12.3	<LOD	<LOD	<LOD	320	1.70	15.0	1.42	<LOD	<LOD	0.14
**GWR8**	**A19**	7.80	539	346	0.02	285	0.90	1.14	0.57	112	20.2	2.03	0.20	0.40	4.68	0.28	0.70	37.1	7.30	<LOD	<LOD	<LOD	470	1.11	10.2	6.00	<LOD	<LOD	0.08
	**S20**	7.90	440	295	1.60	237	1.00	1.27	0.40	92.9	219	1.01	0.40	0.60	2.49	0.33	0.90	53.5	11.9	<LOD	<LOD	<LOD	280	1.60	12.3	5.65	<LOD	<LOD	0.07
	**A20**	8.20	548	342	0.08	278	1.00	1.43	0.55	109	22.2	0.60	0.43	1.77	4.35	0.30	0.97	44.0	10.4	<LOD	<LOD	<LOD	325	1.34	12.0	4.80	<LOD	<LOD	0.13
	**S21**	7.90	539	314	0.47	246	1.27	1.36	0.31	96.2	42.9	1.00	6.25	0.10	7.82	0.29	1.00	68.1	12.9	<LOD	<LOD	<LOD	400	1.26	12.0	3.64	<LOD	<LOD	0.13
**GWR9**	**A19**	7.80	237	134	0.05	124	1.41	3.95	0.65	43.0	3.30	0.80	2.27	1.20	2.25	0.28	1.00	10.5	2.90	<LOD	<LOD	<LOD	146	0.98	12.0	11.0	<LOD	<LOD	0.13
	**S20**	8.10	241	134	2.30	138	0.57	4.31	0.96	48.2	165	2.28	1.43	0.50	2.07	0.39	2.30	16.8	5.08	<LOD	<LOD	<LOD	175	0.88	12.2	9.55	<LOD	<LOD	0.13
	**A20**	7.70	253	134	0.24	111	1.05	4.10	0.68	37.6	16.0	1.30	1.29	0.30	3.28	0.71	5.70	11.8	5.08	<LOD	<LOD	<LOD	170	0.57	1.85	1.30	<LOD	<LOD	0.11
	**S21**	8.00	254	140	0.33	123	0.38	3.59	0.62	43.3	81.3	3.20	0.22	0.31	1.34	0.42	0.84	16.5	6.38	<LOD	<LOD	<LOD	165	0.95	11.8	10.5	<LOD	<LOD	0.07
	GV*	6.5–8.5	2500	500	NA	500	200	NA	NA	NA	200	NA	50	100	70	2000	NA	NA	1300	10	10	3	NA	NA	250	50	3	NA	1.50
	PV**	6.5–9.5	2500	NA	NA	NA	200	NA	NA	NA	200	200	25	50	20	2000	NA	NA	NA	5	10	5	NA	250	250	50	0.50	NA	1.50

* GV-guideline value according to the World Health Organizations Guidelines for Drinking-Water Quality [19], ** PV**-parametric values calculated based on parametric value according to the European Directive 2020/2184 [20].

Table S2. Average Daily Dose (ADD, μg kg^{-1} day^{-1}) of metals and nitrate through the oral and dermal pathways for those in the adult age category exposed to spring waters collected in October 2019 (A19), May 2020 (S20), November 2020 (A20), and Spring 2021 (S21).

Spring	Season	Adults																	
		ADD oral									ADD dermal								
		Fe	Al	Cr	Mn	Ni	Cu	Zn	Ba	NO_3^-	Fe	Al	Cr	Mn	Ni	Cu	Zn	Ba	NO_3^-
**GWR1**	A19	6.35×10^{-1}	1.40×10^{-1}	3.49×10^{-2}	1.89×10^{-2}	8.49×10^{-2}	3.30×10^{-2}	6.29×10^{-2}	2.36×10^{-1}	8.49×10^{1}	2.89×10^{-3}	6.38×10^{-4}	1.59×10^{-4}	8.58×10^{-5}	7.72×10^{-5}	1.50×10^{-4}	1.72×10^{-4}	1.07×10^{-3}	3.99
	S20	5.97	2.28×10^{-1}	5.00×10^{-2}	2.64×10^{-2}	4.87×10^{-2}	1.32×10^{-2}	4.71×10^{-2}	2.11×10^{-1}	5.75×10^{1}	2.72×10^{-2}	1.04×10^{-3}	2.27×10^{-4}	1.20×10^{-4}	4.43×10^{-5}	6.01×10^{-5}	1.29×10^{-4}	9.58×10^{-4}	2.71
	A20	1.91	5.06×10^{-1}	8.14×10^{-2}	5.37×10^{-2}	4.05×10^{-2}	1.79×10^{-2}	2.48×10^{-1}	3.30×10^{-1}	6.07×10^{1}	8.71×10^{-3}	2.30×10^{-3}	3.70×10^{-4}	2.45×10^{-4}	3.69×10^{-5}	8.15×10^{-5}	6.76×10^{-4}	1.50×10^{-3}	2.86
	S21	4.65	1.00	5.41×10^{-2}	6.60×10^{-2}	7.79×10^{-2}	1.19×10^{-2}	5.97×10^{-2}	4.02×10^{-1}	6.47×10^{1}	2.12×10^{-2}	4.56×10^{-3}	2.46×10^{-4}	3.00×10^{-4}	7.09×10^{-5}	5.43×10^{-5}	1.63×10^{-4}	1.83×10^{-3}	3.05
**GWR2**	A19	9.05×10^{-1}	2.69×10^{-1}	6.29×10^{-3}	1.57×10^{-2}	9.40×10^{-2}	1.10×10^{-2}	2.51×10^{-2}	4.62×10^{-1}	1.71×10^{2}	4.12×10^{-3}	1.22×10^{-3}	2.86×10^{-5}	7.15×10^{-5}	8.55×10^{-5}	5.01×10^{-5}	6.86×10^{-5}	2.10×10^{-3}	8.05
	S20	3.17	2.89×10^{-2}	9.43×10^{-3}	1.19×10^{-2}	3.93×10^{-2}	7.86×10^{-3}	3.46×10^{-2}	3.80×10^{-1}	2.16×10^{2}	1.44×10^{-2}	1.32×10^{-4}	4.29×10^{-5}	5.43×10^{-5}	3.58×10^{-5}	3.58×10^{-5}	9.44×10^{-5}	1.73×10^{-3}	1.02×10^{1}
	A20	5.09×10^{-1}	1.79×10^{-1}	1.23×10^{-2}	5.72×10^{-2}	8.36×10^{-2}	1.54×10^{-2}	2.14×10^{-2}	7.26×10^{-1}	2.26×10^{2}	2.32×10^{-3}	8.15×10^{-4}	5.58×10^{-5}	2.60×10^{-4}	7.61×10^{-5}	7.01×10^{-5}	5.83×10^{-5}	3.30×10^{-3}	1.07×10^{1}
	S21	7.51×10^{-1}	2.80×10^{-1}	1.10×10^{-1}	1.26×10^{-2}	1.04×10^{-1}	1.26×10^{-2}	4.71×10^{-2}	5.81×10^{-1}	1.77×10^{2}	3.42×10^{-3}	1.27×10^{-3}	4.99×10^{-4}	5.72×10^{-5}	9.47×10^{-5}	5.72×10^{-5}	1.29×10^{-4}	2.65×10^{-3}	8.33
**GWR3**	A19	8.45×10^{-1}	2.50×10^{-1}	9.43×10^{-3}	1.26×10^{-2}	3.71×10^{-2}	9.43×10^{-3}	2.20×10^{-2}	1.67×10^{-1}	8.01×10^{1}	3.85×10^{-3}	1.14×10^{-3}	4.29×10^{-5}	5.72×10^{-5}	3.38×10^{-5}	4.29×10^{-5}	6.01×10^{-5}	7.58×10^{-4}	3.77
	S20	3.61	3.71×10^{-2}	6.91×10^{-3}	9.43×10^{-3}	4.75×10^{-2}	8.80×10^{-3}	2.51×10^{-2}	3.77×10^{-1}	9.43×10^{1}	1.64×10^{-2}	1.69×10^{-4}	3.15×10^{-5}	4.29×10^{-5}	4.32×10^{-5}	4.00×10^{-5}	6.86×10^{-5}	1.72×10^{-3}	4.44
	A20	4.18×10^{-1}	1.10×10^{-1}	8.17×10^{-3}	4.12×10^{-2}	5.12×10^{-2}	1.10×10^{-2}	1.89×10^{-2}	2.36×10^{-1}	8.11×10^{1}	1.90×10^{-3}	5.01×10^{-4}	3.72×10^{-5}	1.87×10^{-4}	4.66×10^{-5}	5.01×10^{-5}	5.15×10^{-5}	1.08×10^{-3}	3.82
	S21	1.07	4.71×10^{-2}	1.58×10^{-1}	9.43×10^{-3}	1.53×10^{-1}	1.01×10^{-2}	5.03×10^{-2}	5.12×10^{-1}	8.89×10^{1}	4.89×10^{-3}	2.15×10^{-4}	7.21×10^{-4}	4.29×10^{-5}	1.39×10^{-4}	4.58×10^{-5}	1.37×10^{-4}	2.33×10^{-3}	4.19
**GWR5**	A19	4.34×10^{-1}	6.41×10^{-2}	6.29×10^{-3}	9.43×10^{-3}	1.02×10^{-1}	8.80×10^{-3}	2.83×10^{-2}	1.67×10^{-1}	7.51×10^{1}	1.97×10^{-3}	2.92×10^{-4}	2.86×10^{-5}	4.29×10^{-5}	9.32×10^{-5}	4.00×10^{-5}	7.72×10^{-5}	7.58×10^{-4}	3.54
	S20	4.93	2.33×10^{-2}	8.80×10^{-3}	1.13×10^{-2}	7.13×10^{-2}	1.13×10^{-2}	3.14×10^{-2}	2.00×10^{-1}	6.13×10^{1}	2.25×10^{-2}	1.06×10^{-4}	4.00×10^{-5}	5.15×10^{-5}	6.49×10^{-5}	5.15×10^{-5}	8.58×10^{-5}	9.11×10^{-4}	2.88
	A20	6.07×10^{-1}	2.51×10^{-2}	1.79×10^{-2}	4.56×10^{-2}	1.02×10^{-1}	7.86×10^{-3}	2.51×10^{-2}	2.27×10^{-1}	7.35×10^{1}	2.76×10^{-3}	1.14×10^{-4}	8.15×10^{-5}	2.07×10^{-4}	9.27×10^{-5}	3.58×10^{-5}	6.86×10^{-5}	1.03×10^{-3}	3.46
	S21	1.02	9.74×10^{-2}	1.20×10^{-1}	6.29×10^{-3}	1.58×10^{-1}	1.07×10^{-2}	3.46×10^{-2}	2.36×10^{-1}	6.07×10^{1}	4.66×10^{-3}	4.43×10^{-4}	5.45×10^{-4}	2.86×10^{-5}	1.44×10^{-4}	4.86×10^{-5}	9.44×10^{-5}	1.07×10^{-3}	2.86
**GWR6**	A19	7.64×10^{-1}	2.31×10^{-1}	9.43×10^{-3}	1.41×10^{-2}	1.41×10^{-1}	1.07×10^{-2}	2.83×10^{-2}	4.27×10^{-1}	1.85×10^{2}	3.48×10^{-3}	1.05×10^{-3}	4.29×10^{-5}	6.44×10^{-5}	1.28×10^{-4}	4.86×10^{-5}	7.72×10^{-5}	1.94×10^{-3}	8.71
	S20	6.00	5.63×10^{-2}	6.29×10^{-3}	1.01×10^{-2}	7.64×10^{-2}	1.45×10^{-2}	2.20×10^{-2}	4.53×10^{-1}	1.50×10^{2}	2.73×10^{-2}	2.56×10^{-4}	2.86×10^{-5}	4.58×10^{-5}	6.95×10^{-5}	6.58×10^{-5}	6.01×10^{-5}	2.06×10^{-3}	7.04
	A20	7.10×10^{-1}	8.49×10^{-2}	1.51×10^{-2}	1.29×10^{-1}	1.47×10^{-1}	2.01×10^{-2}	3.02×10^{-2}	6.66×10^{-1}	1.10×10^{2}	3.23×10^{-3}	3.86×10^{-4}	6.86×10^{-5}	5.89×10^{-4}	1.34×10^{-4}	9.15×10^{-5}	8.24×10^{-5}	3.03×10^{-3}	5.18
	S21	1.02	1.23×10^{-1}	2.00×10^{-1}	6.29×10^{-3}	2.46×10^{-1}	1.57×10^{-2}	3.14×10^{-2}	7.89×10^{-1}	1.48×10^{2}	4.62×10^{-3}	5.58×10^{-4}	9.08×10^{-4}	2.86×10^{-5}	2.24×10^{-4}	7.15×10^{-5}	8.58×10^{-5}	3.59×10^{-3}	6.95
**GWR7**	A19	7.17×10^{-1}	1.91×10^{-1}	3.14×10^{-2}	6.91×10^{-2}	1.29×10^{-1}	1.63×10^{-2}	2.51×10^{-2}	1.41×10^{-1}	2.73×10^{1}	3.26×10^{-3}	8.68×10^{-4}	1.43×10^{-4}	3.15×10^{-4}	1.18×10^{-4}	7.44×10^{-5}	6.86×10^{-5}	6.44×10^{-4}	1.29
	S20	5.88	3.08×10^{-2}	4.71×10^{-2}	4.87×10^{-1}	8.49×10^{-2}	4.34×10^{-2}	3.46×10^{-2}	2.14×10^{-1}	8.17	2.67×10^{-2}	1.40×10^{-4}	2.15×10^{-4}	2.22×10^{-3}	7.72×10^{-5}	1.97×10^{-4}	9.44×10^{-5}	9.74×10^{-4}	3.85×10^{-1}
	A20	1.52×10^{-1}	1.54×10^{-2}	5.94×10^{-2}	5.25×10^{-2}	1.40×10^{-1}	1.35×10^{-2}	7.07×10^{-2}	3.58×10^{-1}	2.73×10^{2}	6.91×10^{-4}	7.01×10^{-5}	2.70×10^{-4}	2.39×10^{-4}	1.28×10^{-4}	6.15×10^{-5}	1.93×10^{-4}	1.63×10^{-3}	1.29×10^{1}
	S21	9.55×10^{-1}	2.83×10^{-2}	1.73×10^{-1}	5.22×10^{-1}	2.01×10^{-1}	1.95×10^{-2}	4.40×10^{-2}	3.87×10^{-1}	4.46×10^{1}	4.35×10^{-3}	1.29×10^{-4}	7.85×10^{-4}	2.37×10^{-3}	1.83×10^{-4}	8.87×10^{-5}	1.20×10^{-4}	1.76×10^{-3}	2.10
**GWR8**	A19	6.35×10^{-1}	6.38×10^{-2}	6.29×10^{-3}	1.26×10^{-2}	1.47×10^{-1}	8.80×10^{-3}	2.20×10^{-2}	2.29×10^{-1}	1.89×10^{2}	2.89×10^{-3}	2.90×10^{-4}	2.86×10^{-5}	5.72×10^{-5}	1.34×10^{-4}	4.00×10^{-5}	6.01×10^{-5}	1.04×10^{-3}	8.88
	S20	6.88	3.17×10^{-2}	1.26×10^{-2}	1.89×10^{-2}	7.83×10^{-2}	1.04×10^{-2}	2.83×10^{-2}	3.74×10^{-1}	1.78×10^{2}	3.13×10^{-2}	1.44×10^{-4}	5.72×10^{-5}	8.58×10^{-5}	7.12×10^{-5}	4.72×10^{-5}	7.72×10^{-5}	1.70×10^{-3}	8.36
	A20	6.98×10^{-1}	1.89×10^{-2}	1.35×10^{-2}	5.56×10^{-2}	1.37×10^{-1}	9.43×10^{-3}	3.05×10^{-2}	3.27×10^{-1}	1.51×10^{2}	3.17×10^{-3}	8.58×10^{-5}	6.15×10^{-5}	2.53×10^{-4}	1.24×10^{-4}	4.29×10^{-5}	8.32×10^{-5}	1.49×10^{-3}	7.10
	S21	1.35	3.14×10^{-2}	1.96×10^{-1}	3.14×10^{-3}	2.46×10^{-1}	9.11×10^{-3}	3.14×10^{-2}	4.05×10^{-1}	1.14×10^{2}	6.14×10^{-3}	1.43×10^{-4}	8.94×10^{-4}	1.43×10^{-5}	2.24×10^{-4}	4.15×10^{-5}	8.58×10^{-5}	1.84×10^{-3}	5.39
**GWR9**	A19	1.04×10^{-1}	2.51×10^{-2}	7.13×10^{-2}	3.77×10^{-2}	7.07×10^{-2}	8.80×10^{-3}	3.14×10^{-2}	9.11×10^{-2}	3.46×10^{2}	4.72×10^{-4}	1.14×10^{-4}	3.25×10^{-4}	1.72×10^{-4}	6.44×10^{-5}	4.00×10^{-5}	8.58×10^{-5}	4.15×10^{-4}	1.63×10^{1}
	S20	5.19	7.17×10^{-2}	4.49×10^{-2}	1.57×10^{-2}	6.51×10^{-2}	1.23×10^{-2}	7.23×10^{-2}	1.60×10^{-1}	3.00×10^{2}	2.36×10^{-2}	3.26×10^{-4}	2.05×10^{-4}	7.15×10^{-5}	5.92×10^{-5}	5.58×10^{-5}	1.97×10^{-4}	7.27×10^{-4}	1.41×10^{1}
	A20	5.03×10^{-1}	4.09×10^{-2}	4.05×10^{-2}	9.43×10^{-3}	1.03×10^{-1}	2.23×10^{-2}	1.79×10^{-1}	1.60×10^{-1}	4.09×10^{1}	2.29×10^{-3}	1.86×10^{-4}	1.84×10^{-4}	4.29×10^{-5}	9.38×10^{-5}	1.02×10^{-4}	4.89×10^{-4}	7.27×10^{-4}	1.92
	S21	2.56	1.01×10^{-1}	6.91×10^{-3}	9.74×10^{-3}	4.21×10^{-2}	1.32×10^{-2}	2.64×10^{-2}	2.01×10^{-1}	3.30×10^{2}	1.16×10^{-2}	4.58×10^{-4}	3.15×10^{-5}	4.43×10^{-5}	3.83×10^{-5}	6.01×10^{-5}	7.21×10^{-5}	9.12×10^{-4}	1.55×10^{1}

Table S3. Average Daily Dose (ADD, μg kg^{-1} day^{-1}) of metals and nitrate through the oral and dermal pathways for those in the children age category exposed to spring waters collected in October 2019 (A19), May 2020 (S20), November 2020 (A20), and Spring 2021 (S21).

Spring	Season	Children																	
		ADD $_{oral}$									ADD $_{dermal}$								
		Fe	Al	Cr	Mn	Ni	Cu	Zn	Ba	NO_3^-	Fe	Al	Cr	Mn	Ni	Cu	Zn	Ba	NO_3^-
**GWR1**	A19	8.08×10^{-1}	1.78×10^{-1}	4.44×10^{-2}	2.40×10^{-2}	1.08×10^{-1}	4.20×10^{-2}	8.00×10^{-2}	3.00×10^{-1}	1.08×10^{2}	5.11×10^{-3}	1.13×10^{-3}	5.62×10^{-4}	1.52×10^{-4}	1.37×10^{-4}	2.66×10^{-4}	3.04×10^{-4}	1.90×10^{-3}	4.10
	S20	7.60	2.90×10^{-1}	6.36×10^{-2}	3.36×10^{-2}	6.20×10^{-2}	1.68×10^{-2}	6.00×10^{-2}	2.68×10^{-1}	7.32×10^{1}	4.81×10^{-2}	1.84×10^{-3}	8.05×10^{-4}	2.13×10^{-4}	7.85×10^{-5}	1.06×10^{-4}	2.28×10^{-4}	1.70×10^{-3}	2.78
	A20	2.44	6.44×10^{-1}	1.04×10^{-1}	6.84×10^{-2}	5.16×10^{-2}	2.28×10^{-2}	3.15×10^{-1}	4.20×10^{-1}	7.72×10^{1}	1.54×10^{-2}	4.08×10^{-3}	1.31×10^{-3}	4.33×10^{-4}	6.53×10^{-5}	1.44×10^{-4}	1.20×10^{-3}	2.66×10^{-3}	2.93
	S21	5.92	1.28	6.88×10^{-2}	8.40×10^{-2}	9.92×10^{-2}	1.52×10^{-2}	7.60×10^{-2}	5.12×10^{-1}	8.24×10^{1}	3.75×10^{-2}	8.08×10^{-3}	8.71×10^{-4}	5.32×10^{-4}	1.26×10^{-4}	9.62×10^{-5}	2.89×10^{-4}	3.24×10^{-3}	3.13
**GWR2**	A19	1.15	3.42×10^{-1}	8.00×10^{-3}	2.00×10^{-2}	$1.20 \times 10_{-1}$	1.40×10^{-2}	3.20×10^{-2}	5.88×10^{-1}	2.18×10^{2}	7.29×10^{-3}	2.17×10^{-3}	1.01×10^{-4}	1.27×10^{-4}	1.51×10^{-4}	8.86×10^{-5}	1.22×10^{-4}	3.72×10^{-3}	8.26
	S20	4.04	3.68×10^{-2}	1.20×10^{-2}	1.52×10^{-2}	5.00×10^{-2}	1.00×10^{-2}	4.40×10^{-2}	4.84×10^{-1}	2.75×10^{2}	2.56×10^{-2}	2.33×10^{-4}	1.52×10^{-4}	9.62×10^{-5}	6.33×10^{-5}	6.33×10^{-5}	1.67×10^{-4}	3.06×10^{-3}	1.05×10^{1}
	A20	6.48×10^{-1}	2.28×10^{-1}	1.56×10^{-2}	7.28×10^{-2}	1.06×10^{-1}	1.96×10^{-2}	2.72×10^{-2}	9.24×10^{-1}	2.88×10^{2}	4.10×10^{-3}	1.44×10^{-3}	1.97×10^{-4}	4.61×10^{-4}	1.35×10^{-4}	1.24×10^{-4}	1.03×10^{-4}	5.85×10^{-3}	1.09×10^{1}
	S21	$9.56 \times 10_{-1}$	3.56×10^{-1}	1.40×10^{-1}	1.60×10^{-2}	1.32×10^{-1}	1.60×10^{-2}	6.00×10^{-2}	7.40×10^{-1}	2.25×10^{2}	6.05×10^{-3}	2.25×10^{-3}	1.77×10^{-3}	1.01×10^{-4}	1.68×10^{-4}	1.01×10^{-4}	2.28×10^{-4}	4.68×10^{-3}	8.55
**GWR3**	A19	1.08	3.18×10^{-1}	1.20×10^{-2}	1.60×10^{-2}	4.72×10^{-2}	1.20×10^{-2}	2.80×10^{-2}	2.12×10^{-1}	1.02×10^{2}	6.81×10^{-3}	2.01×10^{-3}	1.52×10^{-4}	1.01×10^{-4}	5.97×10^{-5}	7.59×10^{-5}	1.06×10^{-4}	1.34×10^{-3}	3.87
	S20	4.60	4.72×10^{-2}	8.80×10^{-3}	1.20×10^{-2}	6.04×10^{-2}	1.12×10^{-2}	3.20×10^{-2}	4.80×10^{-1}	1.20×10^{2}	2.91×10^{-2}	2.99×10^{-4}	1.11×10^{-4}	7.59×10^{-5}	7.65×10^{-5}	7.09×10^{-5}	1.22×10^{-4}	3.04×10^{-3}	4.56
	A20	5.32×10^{-1}	1.40×10^{-1}	1.04×10^{-2}	5.24×10^{-2}	6.52×10^{-2}	1.40×10^{-2}	2.40×10^{-2}	3.01×10^{-1}	1.03×10^{2}	3.37×10^{-3}	8.86×10^{-4}	1.32×10^{-4}	3.32×10^{-4}	8.25×10^{-5}	8.86×10^{-5}	9.11×10^{-5}	1.90×10^{-3}	3.92
	S21	1.37	6.00×10^{-2}	2.02×10^{-1}	1.20×10^{-2}	1.95×10^{-1}	1.28×10^{-2}	6.40×10^{-2}	6.52×10^{-1}	1.13×10^{2}	8.66×10^{-3}	3.80×10^{-4}	2.55×10^{-3}	7.59×10^{-5}	2.47×10^{-4}	8.10×10^{-5}	2.43×10^{-4}	4.13×10^{-3}	4.30
**GWR5**	A19	5.52×10^{-1}	8.16×10^{-2}	8.00×10^{-3}	1.20×10^{-2}	1.30×10^{-1}	1.12×10^{-2}	3.60×10^{-2}	2.12×10^{-1}	9.56×10^{1}	3.49×10^{-3}	5.16×10^{-4}	1.01×10^{-4}	7.59×10^{-5}	1.65×10^{-4}	7.09×10^{-5}	1.37×10^{-4}	1.34×10^{-3}	3.63
	S20	6.28	2.96×10^{-2}	1.12×10^{-2}	1.44×10^{-2}	9.08×10^{-2}	1.44×10^{-2}	4.00×10^{-2}	2.55×10^{-1}	7.80×10^{1}	3.97×10^{-2}	1.87×10^{-4}	1.42×10^{-4}	9.11×10^{-5}	1.15×10^{-4}	9.11×10^{-5}	1.52×10^{-4}	1.61×10^{-3}	2.96
	A20	7.72×10^{-1}	3.20×10^{-2}	2.28×10^{-2}	5.80×10^{-2}	1.30×10^{-1}	1.00×10^{-2}	3.20×10^{-2}	2.88×10^{-1}	9.36×10^{1}	4.89×10^{-3}	2.03×10^{-4}	2.89×10^{-4}	3.67×10^{-4}	1.64×10^{-4}	6.33×10^{-5}	1.22×10^{-4}	1.83×10^{-3}	3.55
	S21	1.30	1.24×10^{-1}	1.52×10^{-1}	8.00×10^{-3}	2.01×10^{-1}	1.36×10^{-2}	4.40×10^{-2}	3.00×10^{-1}	7.72×10^{1}	8.25×10^{-3}	7.85×10^{-4}	1.93×10^{-3}	5.06×10^{-5}	2.54×10^{-4}	8.61×10^{-5}	1.67×10^{-4}	1.90×10^{-3}	2.93
**GWR6**	A19	9.72×10^{-1}	2.94×10^{-1}	1.20×10^{-2}	1.80×10^{-2}	1.80×10^{-1}	1.36×10^{-2}	3.60×10^{-2}	5.44×10^{-1}	2.36×10^{2}	6.15×10^{-3}	1.86×10^{-3}	1.52×10^{-4}	1.14×10^{-4}	2.27×10^{-4}	8.61×10^{-5}	1.37×10^{-4}	3.44×10^{-3}	8.95
	S20	7.64	7.16×10^{-2}	8.00×10^{-3}	1.28×10^{-2}	9.72×10^{-2}	1.84×10^{-2}	2.80×10^{-2}	5.76×10^{-1}	1.90×10^{2}	4.84×10^{-2}	4.53×10^{-4}	1.01×10^{-4}	8.10×10^{-5}	1.23×10^{-4}	1.16×10^{-4}	1.06×10^{-4}	3.65×10^{-3}	7.23
	A20	9.04×10^{-1}	1.08×10^{-1}	1.92×10^{-2}	1.65×10^{-1}	1.87×10^{-1}	2.56×10^{-2}	3.84×10^{-2}	8.48×10^{-1}	1.40×10^{2}	5.72×10^{-3}	6.84×10^{-4}	2.43×10^{-4}	1.04×10^{-3}	2.37×10^{-4}	1.62×10^{-4}	1.46×10^{-4}	5.37×10^{-3}	5.32
	S21	1.29	1.56×10^{-1}	2.54×10^{-1}	8.00×10^{-3}	3.13×10^{-1}	2.00×10^{-2}	4.00×10^{-2}	1.00	1.88×10^{2}	8.18×10^{-3}	9.87×10^{-4}	3.22×10^{-3}	5.06×10^{-5}	3.96×10^{-4}	1.27×10^{-4}	1.52×10^{-4}	6.35×10^{-3}	7.14
**GWR7**	A19	9.12×10^{-1}	2.43×10^{-1}	4.00×10^{-2}	8.80×10^{-2}	1.64×10^{-1}	2.08×10^{-2}	3.20×10^{-2}	1.80×10^{-1}	3.48×10^{1}	5.77×10^{-3}	1.54×10^{-3}	5.06×10^{-4}	5.57×10^{-4}	2.08×10^{-4}	1.32×10^{-4}	1.22×10^{-4}	1.14×10^{-3}	1.32
	S20	7.48	3.92×10^{-2}	6.00×10^{-2}	6.20×10^{-1}	1.08×10^{-1}	5.52×10^{-2}	4.40×10^{-2}	2.72×10^{-1}	1.04×10^{1}	4.73×10^{-2}	2.48×10^{-4}	7.59×10^{-4}	3.92×10^{-3}	1.37×10^{-4}	3.49×10^{-4}	1.67×10^{-4}	1.72×10^{-3}	3.95×10^{-1}
	A20	1.93×10^{-1}	1.96×10^{-2}	7.56×10^{-2}	6.68×10^{-2}	1.78×10^{-1}	1.72×10^{-2}	9.00×10^{-2}	4.56×10^{-1}	3.48×10^{2}	1.22×10^{-3}	1.24×10^{-4}	9.57×10^{-4}	4.23×10^{-4}	2.26×10^{-4}	1.09×10^{-4}	3.42×10^{-4}	2.89×10^{-3}	1.32×10^{1}
	S21	1.22	3.60×10^{-2}	2.20×10^{-1}	6.64×10^{-1}	2.56×10^{-1}	2.48×10^{-2}	5.60×10^{-2}	4.92×10^{-1}	5.68×10^{1}	7.70×10^{-3}	2.28×10^{-4}	2.78×10^{-3}	4.20×10^{-3}	3.25×10^{-4}	1.57×10^{-4}	2.13×10^{-4}	3.11×10^{-3}	2.16
**GWR8**	A19	8.08×10^{-1}	8.12×10^{-2}	8.00×10^{-3}	1.60×10^{-2}	1.87×10^{-1}	1.12×10^{-2}	2.80×10^{-2}	2.92×10^{-1}	2.40×10^{2}	5.11×10^{-3}	5.14×10^{-4}	1.01×10^{-4}	1.01×10^{-4}	2.37×10^{-4}	7.09×10^{-5}	1.06×10^{-4}	1.85×10^{-3}	9.11
	S20	8.76	4.04×10^{-2}	1.60×10^{-2}	2.40×10^{-2}	9.96×10^{-2}	1.32×10^{-2}	3.60×10^{-2}	4.76×10^{-1}	2.26×10^{2}	5.54×10^{-2}	2.56×10^{-4}	2.03×10^{-4}	1.52×10^{-4}	1.26×10^{-4}	8.35×10^{-5}	1.37×10^{-4}	3.01×10^{-3}	8.58
	A20	8.88×10^{-1}	2.40×10^{-2}	1.72×10^{-2}	7.08×10^{-2}	1.74×10^{-1}	1.20×10^{-2}	3.88×10^{-2}	4.16×10^{-1}	1.92×10^{2}	5.62×10^{-3}	1.52×10^{-4}	2.18×10^{-4}	4.48×10^{-4}	2.20×10^{-4}	7.59×10^{-5}	1.47×10^{-4}	2.63×10^{-3}	7.29
	S21	1.72	4.00×10^{-2}	2.50×10^{-1}	4.00×10^{-3}	3.13×10^{-1}	1.16×10^{-2}	4.00×10^{-2}	5.16×10^{-1}	1.46×10^{2}	1.09×10^{-2}	2.53×10^{-4}	3.16×10^{-4}	2.53×10^{-5}	3.96×10^{-4}	7.34×10^{-5}	1.52×10^{-4}	3.27×10^{-3}	5.53
**GWR9**	A19	1.32×10^{-1}	3.20×10^{-2}	9.08×10^{-2}	4.80×10^{-2}	9.00×10^{-2}	1.12×10^{-2}	4.00×10^{-2}	1.16×10^{-1}	4.40×10^{2}	8.35×10^{-4}	2.03×10^{-4}	1.15×10^{-3}	3.04×10^{-4}	1.14×10^{-4}	7.09×10^{-5}	1.52×10^{-4}	7.34×10^{-4}	1.67×10^{1}
	S20	6.60	9.12×10^{-2}	5.72×10^{-2}	2.00×10^{-2}	8.28×10^{-2}	1.56×10^{-2}	9.20×10^{-2}	2.03×10^{-1}	3.82×10^{2}	4.18×10^{-2}	5.77×10^{-4}	7.24×10^{-4}	1.27×10^{-4}	1.05×10^{-4}	9.87×10^{-5}	3.49×10^{-4}	1.29×10^{-3}	1.45×10^{1}
	A20	6.40×10^{-1}	5.20×10^{-2}	5.16×10^{-2}	1.20×10^{-2}	1.31×10^{-1}	2.84×10^{-2}	2.28×10^{-1}	2.03×10^{-1}	5.20×10^{1}	4.05×10^{-3}	3.29×10^{-4}	6.53×10^{-4}	7.59×10^{-5}	1.66×10^{-4}	1.80×10^{-4}	8.66×10^{-4}	1.29×10^{-3}	1.97
	S21	3.25	1.28×10^{-1}	8.80×10^{-3}	1.24×10^{-2}	5.36×10^{-2}	1.68×10^{-2}	3.36×10^{-2}	2.55×10^{-1}	4.20×10^{2}	2.06×10^{-2}	8.10×10^{-4}	1.11×10^{-4}	7.85×10^{-5}	6.78×10^{-5}	1.06×10^{-4}	1.28×10^{-4}	1.62×10^{-3}	1.59×10^{1}

Table S4. Hazard Quotient (HQ) values of metals and nitrate through the oral and dermal pathways for those in the adult age category exposed to spring waters collected in October 2019 (A19), May 2020 (S20), November 2020 (A20), and Spring 2021 (S21).

Spring	Season	Adult																	
		HQ oral									HQ dermal								
		Fe	Al	Cr	Mn	Ni	Cu	Zn	Ba	NO_3^-	Fe	Al	Cr	Mn	Ni	Cu	Zn	Ba	NO_3^-
**GWR1**	A19	9.07×10^{-4}	1.40×10^{-4}	1.16×10^{-2}	7.86×10^{-4}	4.24×10^{-3}	8.25×10^{-4}	2.10×10^{-4}	1.18×10^{-3}	5.30×10^{-2}	2.06×10^{-5}	3.19×10^{-6}	2.12×10^{-3}	8.94×10^{-5}	9.65×10^{-5}	1.88×10^{-5}	2.86×10^{-6}	7.66×10^{-5}	2.50×10^{-3}
	S20	8.53×10^{-3}	2.28×10^{-4}	1.67×10^{-2}	1.10×10^{-3}	2.44×10^{-3}	3.30×10^{-4}	1.57×10^{-4}	1.05×10^{-3}	3.59×10^{-2}	1.94×10^{-4}	5.18×10^{-6}	3.03×10^{-3}	1.25×10^{-4}	5.54×10^{-5}	7.51×10^{-6}	2.15×10^{-6}	6.84×10^{-5}	1.69×10^{-3}
	A20	2.73×10^{-3}	5.06×10^{-4}	2.71×10^{-2}	2.24×10^{-3}	2.03×10^{-3}	4.48×10^{-4}	8.26×10^{-4}	1.65×10^{-3}	3.79×10^{-2}	6.22×10^{-5}	1.15×10^{-5}	4.94×10^{-3}	2.55×10^{-4}	4.61×10^{-5}	1.02×10^{-5}	1.13×10^{-5}	1.07×10^{-4}	1.78×10^{-3}
	S21	6.64×10^{-3}	1.00×10^{-3}	1.80×10^{-2}	2.75×10^{-3}	3.90×10^{-3}	2.99×10^{-4}	1.99×10^{-4}	2.01×10^{-3}	4.05×10^{-2}	1.51×10^{-4}	2.28×10^{-5}	3.28×10^{-3}	3.13×10^{-4}	8.87×10^{-5}	6.79×10^{-6}	2.72×10^{-6}	1.31×10^{-4}	1.90×10^{-3}
**GWR2**	A19	1.29×10^{-3}	2.69×10^{-4}	2.10×10^{-3}	6.55×10^{-4}	4.70×10^{-3}	2.75×10^{-4}	8.38×10^{-5}	2.31×10^{-3}	1.07×10^{-1}	2.94×10^{-5}	6.12×10^{-6}	3.81×10^{-4}	7.45×10^{-5}	1.07×10^{-4}	6.26×10^{-6}	1.14×10^{-6}	1.50×10^{-4}	5.03×10^{-3}
	S20	4.53×10^{-3}	2.89×10^{-5}	3.14×10^{-3}	4.98×10^{-4}	1.96×10^{-3}	1.96×10^{-4}	1.15×10^{-4}	1.90×10^{-3}	1.35×10^{-1}	1.03×10^{-4}	6.58×10^{-7}	5.72×10^{-4}	5.66×10^{-5}	4.47×10^{-5}	4.47×10^{-6}	1.57×10^{-6}	1.24×10^{-4}	6.36×10^{-3}
	A20	7.27×10^{-4}	1.79×10^{-4}	4.09×10^{-3}	2.38×10^{-3}	4.18×10^{-3}	3.85×10^{-4}	7.12×10^{-5}	3.63×10^{-3}	1.41×10^{-1}	1.65×10^{-5}	4.08×10^{-6}	7.44×10^{-4}	2.71×10^{-4}	9.51×10^{-5}	8.76×10^{-6}	9.72×10^{-7}	2.36×10^{-4}	6.66×10^{-3}
	S21	1.07×10^{-3}	2.80×10^{-4}	3.66×10^{-2}	5.24×10^{-4}	5.20×10^{-3}	3.14×10^{-4}	1.57×10^{-4}	2.91×10^{-3}	1.11×10^{-1}	2.44×10^{-5}	6.36×10^{-6}	6.65×10^{-3}	5.96×10^{-5}	1.18×10^{-4}	7.15×10^{-6}	2.15×10^{-6}	1.89×10^{-4}	5.21×10^{-3}
**GWR3**	A19	1.21×10^{-3}	2.50×10^{-4}	3.14×10^{-3}	5.24×10^{-4}	1.85×10^{-3}	2.36×10^{-4}	7.33×10^{-5}	8.33×10^{-4}	5.01×10^{-2}	2.75×10^{-5}	5.68×10^{-6}	5.72×10^{-4}	5.96×10^{-5}	4.22×10^{-5}	5.36×10^{-6}	1.00×10^{-6}	5.41×10^{-5}	2.36×10^{-3}
	S20	5.16×10^{-3}	3.71×10^{-5}	2.30×10^{-3}	3.93×10^{-4}	2.37×10^{-3}	2.20×10^{-4}	8.38×10^{-5}	1.89×10^{-3}	5.89×10^{-2}	1.17×10^{-4}	8.44×10^{-7}	4.20×10^{-4}	4.47×10^{-5}	5.40×10^{-5}	5.01×10^{-6}	1.14×10^{-6}	1.23×10^{-4}	2.77×10^{-3}
	A20	5.97×10^{-4}	1.10×10^{-4}	2.72×10^{-3}	1.72×10^{-3}	2.56×10^{-3}	2.75×10^{-4}	6.29×10^{-5}	1.18×10^{-3}	5.07×10^{-2}	1.36×10^{-5}	2.50×10^{-6}	4.96×10^{-4}	1.95×10^{-4}	5.83×10^{-5}	6.26×10^{-6}	8.58×10^{-7}	7.68×10^{-5}	2.39×10^{-3}
	S21	1.54×10^{-3}	4.71×10^{-5}	5.28×10^{-2}	3.93×10^{-4}	7.65×10^{-3}	2.51×10^{-4}	1.68×10^{-4}	2.56×10^{-3}	5.56×10^{-2}	3.49×10^{-5}	1.07×10^{-6}	9.61×10^{-3}	4.47×10^{-5}	1.74×10^{-4}	5.72×10^{-6}	2.29×10^{-6}	1.67×10^{-4}	2.62×10^{-3}
**GWR5**	A19	6.20×10^{-4}	6.41×10^{-5}	2.10×10^{-3}	3.93×10^{-4}	5.12×10^{-3}	2.20×10^{-4}	9.43×10^{-5}	8.33×10^{-4}	4.69×10^{-2}	1.41×10^{-5}	1.46×10^{-6}	3.81×10^{-4}	4.47×10^{-5}	1.17×10^{-4}	5.01×10^{-6}	1.29×10^{-6}	5.41×10^{-5}	2.21×10^{-3}
	S20	7.05×10^{-3}	2.33×10^{-5}	2.93×10^{-3}	4.71×10^{-4}	3.57×10^{-3}	2.83×10^{-4}	1.05×10^{-4}	1.00×10^{-3}	3.83×10^{-2}	1.60×10^{-4}	5.29×10^{-7}	5.34×10^{-4}	5.36×10^{-5}	8.12×10^{-5}	6.44×10^{-6}	1.43×10^{-6}	6.51×10^{-5}	1.80×10^{-3}
	A20	8.67×10^{-4}	2.51×10^{-5}	5.97×10^{-3}	1.90×10^{-3}	5.09×10^{-3}	1.96×10^{-4}	8.38×10^{-5}	1.13×10^{-3}	4.60×10^{-2}	1.97×10^{-5}	5.72×10^{-7}	1.09×10^{-3}	2.16×10^{-4}	1.16×10^{-4}	4.47×10^{-6}	1.14×10^{-6}	7.37×10^{-5}	2.16×10^{-3}
	S21	1.46×10^{-3}	9.74×10^{-5}	3.99×10^{-2}	2.62×10^{-4}	7.89×10^{-3}	2.67×10^{-4}	1.15×10^{-4}	1.18×10^{-3}	3.79×10^{-2}	3.33×10^{-5}	2.22×10^{-6}	7.27×10^{-3}	2.98×10^{-5}	1.79×10^{-4}	6.08×10^{-6}	1.57×10^{-6}	7.66×10^{-5}	1.78×10^{-3}
**GWR6**	A19	1.09×10^{-3}	2.31×10^{-4}	3.14×10^{-3}	5.89×10^{-4}	7.06×10^{-3}	2.67×10^{-4}	9.43×10^{-5}	2.14×10^{-3}	1.16×10^{-1}	2.48×10^{-5}	5.25×10^{-6}	5.72×10^{-4}	6.70×10^{-5}	1.61×10^{-4}	6.08×10^{-6}	1.29×10^{-6}	1.39×10^{-4}	5.45×10^{-3}
	S20	8.58×10^{-3}	5.63×10^{-5}	2.10×10^{-3}	4.19×10^{-4}	3.82×10^{-3}	3.61×10^{-4}	7.33×10^{-5}	2.26×10^{-3}	9.35×10^{-2}	1.95×10^{-4}	1.28×10^{-6}	3.81×10^{-4}	4.77×10^{-5}	8.69×10^{-5}	8.22×10^{-6}	1.00×10^{-6}	1.47×10^{-4}	4.40×10^{-3}
	A20	1.01×10^{-3}	8.49×10^{-5}	5.03×10^{-3}	5.40×10^{-3}	7.35×10^{-3}	5.03×10^{-4}	1.01×10^{-4}	3.33×10^{-3}	6.88×10^{-2}	2.31×10^{-5}	1.93×10^{-6}	9.15×10^{-4}	6.14×10^{-4}	1.67×10^{-4}	1.14×10^{-5}	1.37×10^{-6}	2.17×10^{-4}	3.24×10^{-3}
	S21	1.45×10^{-3}	1.23×10^{-4}	6.65×10^{-2}	2.62×10^{-4}	1.23×10^{-2}	3.93×10^{-4}	1.05×10^{-4}	3.94×10^{-3}	9.23×10^{-2}	3.30×10^{-5}	2.79×10^{-6}	1.21×10^{-2}	2.98×10^{-5}	2.80×10^{-4}	8.94×10^{-6}	1.43×10^{-6}	2.56×10^{-4}	4.35×10^{-3}
**GWR7**	A19	1.02×10^{-3}	1.91×10^{-4}	1.05×10^{-2}	2.88×10^{-3}	6.46×10^{-3}	4.09×10^{-4}	8.38×10^{-5}	7.07×10^{-4}	1.71×10^{-2}	2.33×10^{-5}	4.34×10^{-6}	1.91×10^{-3}	3.28×10^{-4}	1.47×10^{-4}	9.30×10^{-6}	1.14×10^{-6}	4.60×10^{-5}	8.04×10^{-4}
	S20	8.40×10^{-3}	3.08×10^{-5}	1.57×10^{-2}	2.03×10^{-2}	4.24×10^{-3}	1.08×10^{-3}	1.15×10^{-4}	1.07×10^{-3}	5.11×10^{-3}	1.91×10^{-4}	7.01×10^{-7}	2.86×10^{-3}	2.31×10^{-3}	9.65×10^{-5}	2.47×10^{-5}	1.57×10^{-6}	6.96×10^{-5}	2.40×10^{-4}
	A20	2.17×10^{-4}	1.54×10^{-5}	1.98×10^{-2}	2.19×10^{-3}	7.01×10^{-3}	3.38×10^{-4}	2.36×10^{-4}	1.79×10^{-3}	1.71×10^{-1}	4.93×10^{-6}	3.50×10^{-7}	3.60×10^{-3}	2.49×10^{-4}	1.59×10^{-4}	7.69×10^{-6}	3.22×10^{-6}	1.16×10^{-4}	8.04×10^{-3}
	S21	1.36×10^{-3}	2.83×10^{-5}	5.75×10^{-2}	2.17×10^{-2}	1.01×10^{-2}	4.87×10^{-4}	1.47×10^{-4}	1.93×10^{-3}	2.79×10^{-2}	3.11×10^{-5}	6.44×10^{-7}	1.05×10^{-2}	2.47×10^{-3}	2.29×10^{-4}	1.11×10^{-5}	2.00×10^{-6}	1.26×10^{-4}	1.31×10^{-3}
**GWR8**	A19	9.07×10^{-4}	6.38×10^{-5}	2.10×10^{-3}	5.24×10^{-4}	7.35×10^{-3}	2.20×10^{-4}	7.33×10^{-5}	1.15×10^{-3}	1.18×10^{-1}	2.06×10^{-5}	1.45×10^{-6}	3.81×10^{-4}	5.96×10^{-5}	1.67×10^{-4}	5.01×10^{-6}	1.00×10^{-6}	7.46×10^{-5}	5.55×10^{-3}
	S20	9.83×10^{-3}	3.17×10^{-5}	4.19×10^{-3}	7.86×10^{-4}	3.91×10^{-3}	2.59×10^{-4}	9.43×10^{-5}	1.87×10^{-3}	1.11×10^{-1}	2.24×10^{-4}	7.22×10^{-7}	7.63×10^{-4}	8.94×10^{-5}	8.90×10^{-5}	5.90×10^{-6}	1.29×10^{-6}	1.22×10^{-4}	5.22×10^{-3}
	A20	9.97×10^{-4}	1.89×10^{-5}	4.50×10^{-3}	2.32×10^{-3}	6.84×10^{-3}	2.36×10^{-4}	1.02×10^{-4}	1.63×10^{-3}	9.43×10^{-2}	2.27×10^{-5}	4.29×10^{-7}	8.20×10^{-4}	2.64×10^{-4}	1.56×10^{-4}	5.36×10^{-6}	1.39×10^{-6}	1.06×10^{-4}	4.44×10^{-3}
	S21	1.93×10^{-3}	3.14×10^{-5}	6.55×10^{-2}	1.31×10^{-4}	1.23×10^{-2}	2.28×10^{-4}	1.05×10^{-4}	2.03×10^{-3}	7.15×10^{-2}	4.38×10^{-5}	7.15×10^{-7}	1.19×10^{-2}	1.49×10^{-5}	2.80×10^{-4}	5.18×10^{-6}	1.43×10^{-6}	1.32×10^{-4}	3.37×10^{-3}
**GWR9**	A19	1.48×10^{-4}	2.51×10^{-5}	2.38×10^{-2}	1.57×10^{-3}	3.54×10^{-3}	2.20×10^{-4}	1.05×10^{-4}	4.56×10^{-4}	2.16×10^{-1}	3.37×10^{-6}	5.72×10^{-7}	4.33×10^{-3}	1.79×10^{-4}	8.04×10^{-5}	5.01×10^{-6}	1.43×10^{-6}	2.96×10^{-5}	1.02×10^{-2}
	S20	7.41×10^{-3}	7.17×10^{-5}	1.50×10^{-2}	6.55×10^{-4}	3.25×10^{-3}	3.06×10^{-4}	2.41×10^{-4}	7.98×10^{-4}	1.88×10^{-1}	1.69×10^{-4}	1.63×10^{-6}	2.73×10^{-3}	7.45×10^{-5}	7.40×10^{-5}	6.97×10^{-6}	3.29×10^{-6}	5.19×10^{-5}	8.83×10^{-3}
	A20	7.18×10^{-4}	4.09×10^{-5}	1.35×10^{-2}	3.93×10^{-4}	5.15×10^{-3}	5.58×10^{-4}	5.97×10^{-4}	7.98×10^{-4}	2.55×10^{-2}	1.63×10^{-5}	9.30×10^{-7}	2.46×10^{-3}	4.47×10^{-5}	1.17×10^{-4}	1.27×10^{-5}	8.15×10^{-6}	5.19×10^{-5}	1.20×10^{-3}
	S21	3.65×10^{-3}	1.01×10^{-4}	2.30×10^{-3}	4.06×10^{-4}	2.11×10^{-3}	3.30×10^{-4}	8.80×10^{-5}	1.00×10^{-3}	2.06×10^{-1}	8.31×10^{-5}	2.29×10^{-6}	4.20×10^{-4}	4.62×10^{-5}	4.79×10^{-5}	7.51×10^{-6}	1.20×10^{-6}	6.52×10^{-5}	9.71×10^{-3}

Table S5. Hazard Quotient (HQ) of metals and nitrate through the oral and dermal pathways for those in the children age category exposed to spring waters collected in October 2019 (A19), May 2020 (S20), November 2020 (A20), and Spring 2021 (S21).

Spring	Season	Children																	
		HQ_{oral}									HQ_{dermal}								
		Fe	Al	Cr	Mn	Ni	Cu	Zn	Ba	NO_3^-	Fe	Al	Cr	Mn	Ni	Cu	Zn	Ba	NO_3^-
**GWR1**	A19	1.15×10^{-3}	1.78×10^{-4}	1.48×10^{-2}	1.00×10^{-3}	5.40×10^{-3}	1.05×10^{-3}	2.67×10^{-4}	1.50×10^{-3}	6.75×10^{-2}	3.65×10^{-5}	5.65×10^{-6}	7.49×10^{-3}	1.58×10^{-4}	1.71×10^{-4}	3.32×10^{-5}	5.06×10^{-6}	1.36×10^{-4}	2.56×10^{-3}
	S20	1.09×10^{-2}	2.90×10^{-4}	2.12×10^{-2}	1.40×10^{-3}	3.10×10^{-3}	4.20×10^{-4}	2.00×10^{-4}	1.34×10^{-3}	4.58×10^{-2}	3.44×10^{-4}	9.18×10^{-6}	1.07×10^{-2}	2.22×10^{-4}	9.81×10^{-5}	1.33×10^{-5}	3.80×10^{-6}	1.21×10^{-4}	1.74×10^{-3}
	A20	3.48×10^{-3}	6.44×10^{-4}	3.45×10^{-2}	2.85×10^{-3}	2.58×10^{-3}	5.70×10^{-4}	1.05×10^{-3}	2.10×10^{-3}	4.83×10^{-2}	1.10×10^{-4}	2.04×10^{-5}	1.75×10^{-2}	4.51×10^{-4}	8.16×10^{-5}	1.80×10^{-5}	1.99×10^{-5}	1.90×10^{-4}	1.83×10^{-3}
	S21	8.46×10^{-3}	1.28×10^{-3}	2.29×10^{-2}	3.50×10^{-3}	4.96×10^{-3}	3.80×10^{-4}	2.53×10^{-4}	2.56×10^{-3}	5.15×10^{-2}	2.68×10^{-4}	4.04×10^{-5}	1.16×10^{-2}	5.54×10^{-4}	1.57×10^{-4}	1.20×10^{-5}	4.81×10^{-6}	2.31×10^{-4}	1.96×10^{-3}
**GWR2**	A19	1.65×10^{-3}	3.42×10^{-4}	2.67×10^{-3}	8.33×10^{-4}	5.98×10^{-3}	3.50×10^{-4}	1.07×10^{-4}	2.94×10^{-3}	1.36×10^{-1}	5.21×10^{-5}	1.08×10^{-5}	1.35×10^{-3}	1.32×10^{-4}	1.89×10^{-4}	1.11×10^{-5}	2.03×10^{-6}	2.66×10^{-4}	5.16×10^{-3}
	S20	5.77×10^{-3}	3.68×10^{-5}	4.00×10^{-3}	6.33×10^{-4}	2.50×10^{-3}	2.50×10^{-4}	1.47×10^{-4}	2.42×10^{-3}	1.72×10^{-1}	1.83×10^{-4}	1.16×10^{-6}	2.03×10^{-3}	1.00×10^{-4}	7.91×10^{-5}	7.91×10^{-6}	2.78×10^{-6}	2.19×10^{-4}	6.53×10^{-3}
	A20	9.26×10^{-4}	2.28×10^{-4}	5.20×10^{-3}	3.03×10^{-3}	5.32×10^{-3}	4.90×10^{-4}	9.07×10^{-5}	4.62×10^{-3}	1.80×10^{-1}	2.93×10^{-5}	7.21×10^{-6}	2.63×10^{-3}	4.80×10^{-4}	1.68×10^{-4}	1.55×10^{-5}	1.72×10^{-6}	4.18×10^{-4}	6.84×10^{-3}
	S21	1.37×10^{-3}	3.56×10^{-4}	4.65×10^{-2}	6.67×10^{-4}	6.62×10^{-3}	4.00×10^{-4}	2.00×10^{-4}	3.70×10^{-3}	1.41×10^{-1}	4.32×10^{-5}	1.13×10^{-5}	2.36×10^{-2}	1.05×10^{-4}	2.09×10^{-4}	1.27×10^{-5}	3.80×10^{-6}	3.35×10^{-4}	5.34×10^{-3}
**GWR3**	A19	1.54×10^{-3}	3.18×10^{-4}	4.00×10^{-3}	6.67×10^{-4}	2.36×10^{-3}	3.00×10^{-4}	9.33×10^{-5}	1.06×10^{-3}	6.38×10^{-2}	4.86×10^{-5}	1.01×10^{-5}	2.03×10^{-3}	1.05×10^{-4}	7.47×10^{-5}	9.49×10^{-6}	1.77×10^{-6}	9.58×10^{-5}	2.42×10^{-3}
	S20	6.57×10^{-3}	4.72×10^{-5}	2.93×10^{-3}	5.00×10^{-4}	3.02×10^{-3}	2.80×10^{-4}	1.07×10^{-4}	2.40×10^{-3}	7.50×10^{-2}	2.08×10^{-4}	1.49×10^{-6}	1.49×10^{-3}	7.91×10^{-5}	9.56×10^{-5}	8.86×10^{-6}	2.03×10^{-6}	2.17×10^{-4}	2.85×10^{-3}
	A20	7.60×10^{-4}	1.40×10^{-4}	3.47×10^{-3}	2.18×10^{-3}	3.26×10^{-3}	3.50×10^{-4}	8.00×10^{-5}	1.50×10^{-3}	6.45×10^{-2}	2.40×10^{-5}	4.43×10^{-6}	1.76×10^{-3}	3.45×10^{-4}	1.03×10^{-4}	1.11×10^{-5}	1.52×10^{-6}	1.36×10^{-4}	2.45×10^{-3}
	S21	1.95×10^{-3}	6.00×10^{-5}	6.72×10^{-2}	5.00×10^{-4}	9.74×10^{-3}	3.20×10^{-4}	2.13×10^{-4}	3.26×10^{-3}	7.08×10^{-2}	6.18×10^{-5}	1.90×10^{-6}	3.40×10^{-2}	7.91×10^{-5}	3.08×10^{-4}	1.01×10^{-5}	4.05×10^{-6}	2.95×10^{-4}	2.69×10^{-3}
**GWR5**	A19	7.89×10^{-4}	8.16×10^{-5}	2.67×10^{-3}	5.00×10^{-4}	6.52×10^{-3}	2.80×10^{-4}	1.20×10^{-4}	1.06×10^{-3}	5.98×10^{-2}	2.50×10^{-5}	2.58×10^{-6}	1.35×10^{-3}	7.91×10^{-5}	2.06×10^{-4}	8.86×10^{-6}	2.28×10^{-6}	9.58×10^{-5}	2.27×10^{-3}
	S20	8.97×10^{-3}	2.96×10^{-5}	3.73×10^{-3}	6.00×10^{-4}	4.54×10^{-3}	3.60×10^{-4}	1.33×10^{-4}	1.27×10^{-3}	4.88×10^{-2}	2.84×10^{-4}	9.37×10^{-7}	1.89×10^{-3}	9.49×10^{-5}	1.44×10^{-4}	1.14×10^{-5}	2.53×10^{-6}	1.15×10^{-4}	1.85×10^{-3}
	A20	1.10×10^{-3}	3.20×10^{-5}	7.60×10^{-3}	2.42×10^{-3}	6.48×10^{-3}	2.50×10^{-4}	1.07×10^{-4}	1.44×10^{-3}	5.85×10^{-2}	3.49×10^{-5}	1.01×10^{-6}	3.85×10^{-3}	3.82×10^{-4}	2.05×10^{-4}	7.91×10^{-6}	2.03×10^{-6}	1.30×10^{-4}	2.22×10^{-3}
	S21	1.86×10^{-3}	1.24×10^{-4}	5.08×10^{-2}	3.33×10^{-4}	1.00×10^{-2}	3.40×10^{-4}	1.47×10^{-4}	1.50×10^{-3}	4.83×10^{-2}	5.89×10^{-5}	3.92×10^{-6}	2.57×10^{-2}	5.27×10^{-5}	3.18×10^{-4}	1.08×10^{-5}	2.78×10^{-6}	1.36×10^{-4}	1.83×10^{-3}
**GWR6**	A19	1.39×10^{-3}	2.94×10^{-4}	4.00×10^{-3}	7.50×10^{-4}	8.98×10^{-3}	3.40×10^{-4}	1.20×10^{-4}	2.72×10^{-3}	1.47×10^{-1}	4.39×10^{-5}	9.29×10^{-6}	2.03×10^{-3}	1.19×10^{-4}	2.84×10^{-4}	1.08×10^{-5}	2.28×10^{-6}	2.46×10^{-4}	5.59×10^{-3}
	S20	1.09×10^{-2}	7.16×10^{-5}	2.67×10^{-3}	5.33×10^{-4}	4.86×10^{-3}	4.60×10^{-4}	9.33×10^{-5}	2.88×10^{-3}	1.19×10^{-1}	3.45×10^{-4}	2.27×10^{-6}	1.35×10^{-3}	8.44×10^{-5}	1.54×10^{-4}	1.46×10^{-5}	1.77×10^{-6}	2.60×10^{-4}	4.52×10^{-3}
	A20	1.29×10^{-3}	1.08×10^{-4}	6.40×10^{-3}	6.87×10^{-3}	9.36×10^{-3}	6.40×10^{-4}	1.28×10^{-4}	4.24×10^{-3}	8.75×10^{-2}	4.09×10^{-5}	3.42×10^{-6}	3.24×10^{-3}	1.09×10^{-3}	2.96×10^{-4}	2.03×10^{-5}	2.43×10^{-6}	3.83×10^{-4}	3.32×10^{-3}
	S21	1.85×10^{-3}	1.56×10^{-4}	8.47×10^{-2}	3.33×10^{-4}	1.56×10^{-2}	5.00×10^{-4}	1.33×10^{-4}	5.02×10^{-3}	1.18×10^{-1}	5.84×10^{-5}	4.94×10^{-6}	4.29×10^{-2}	5.27×10^{-5}	4.95×10^{-4}	1.58×10^{-5}	2.53×10^{-6}	4.54×10^{-4}	4.46×10^{-3}
**GWR7**	A19	1.30×10^{-3}	2.43×10^{-4}	1.33×10^{-2}	3.67×10^{-3}	8.22×10^{-3}	5.20×10^{-4}	1.07×10^{-4}	9.00×10^{-4}	2.18×10^{-2}	4.12×10^{-5}	7.68×10^{-6}	6.75×10^{-3}	5.80×10^{-4}	2.60×10^{-4}	1.65×10^{-5}	2.03×10^{-6}	8.14×10^{-5}	8.26×10^{-4}
	S20	1.07×10^{-2}	3.92×10^{-5}	2.00×10^{-2}	2.58×10^{-2}	5.40×10^{-3}	1.38×10^{-3}	1.47×10^{-4}	1.36×10^{-3}	6.50×10^{-3}	3.38×10^{-4}	1.24×10^{-6}	1.01×10^{-2}	4.09×10^{-3}	1.71×10^{-4}	4.37×10^{-5}	2.78×10^{-6}	1.23×10^{-4}	2.47×10^{-4}
	A20	2.76×10^{-4}	1.96×10^{-5}	2.52×10^{-2}	2.78×10^{-3}	8.92×10^{-3}	4.30×10^{-4}	3.00×10^{-4}	2.28×10^{-3}	2.17×10^{-1}	8.73×10^{-6}	6.20×10^{-7}	1.28×10^{-2}	4.40×10^{-4}	2.82×10^{-4}	1.36×10^{-5}	5.70×10^{-6}	2.06×10^{-4}	8.25×10^{-3}
	S21	1.74×10^{-3}	3.60×10^{-5}	7.32×10^{-2}	2.77×10^{-2}	1.28×10^{-2}	6.20×10^{-4}	1.87×10^{-4}	2.46×10^{-3}	3.55×10^{-2}	5.50×10^{-5}	1.14×10^{-6}	3.71×10^{-2}	4.38×10^{-3}	4.06×10^{-4}	1.96×10^{-5}	3.54×10^{-6}	2.22×10^{-4}	1.35×10^{-3}
**GWR8**	A19	1.15×10^{-3}	8.12×10^{-5}	2.67×10^{-3}	6.67×10^{-4}	9.36×10^{-3}	2.80×10^{-4}	9.33×10^{-5}	1.46×10^{-3}	1.50×10^{-1}	3.65×10^{-5}	2.57×10^{-6}	1.35×10^{-3}	1.05×10^{-4}	2.96×10^{-4}	8.86×10^{-6}	1.77×10^{-6}	1.32×10^{-4}	5.70×10^{-3}
	S20	1.25×10^{-2}	4.04×10^{-5}	5.33×10^{-3}	1.00×10^{-3}	4.98×10^{-3}	3.30×10^{-4}	1.20×10^{-4}	2.38×10^{-3}	1.41×10^{-1}	3.96×10^{-4}	1.28×10^{-6}	2.70×10^{-3}	1.58×10^{-4}	1.58×10^{-4}	1.04×10^{-5}	2.28×10^{-6}	2.15×10^{-4}	5.36×10^{-3}
	A20	1.27×10^{-3}	2.40×10^{-5}	5.73×10^{-3}	2.95×10^{-3}	8.70×10^{-3}	3.00×10^{-4}	1.29×10^{-4}	2.08×10^{-3}	1.20×10^{-1}	4.01×10^{-5}	7.59×10^{-7}	2.90×10^{-3}	4.67×10^{-4}	2.75×10^{-4}	9.49×10^{-6}	2.46×10^{-6}	1.88×10^{-4}	4.56×10^{-3}
	S21	2.45×10^{-3}	4.00×10^{-5}	8.33×10^{-2}	1.67×10^{-4}	1.56×10^{-2}	2.90×10^{-4}	1.33×10^{-4}	2.58×10^{-3}	9.10×10^{-2}	7.76×10^{-5}	1.27×10^{-6}	4.22×10^{-2}	2.64×10^{-5}	4.95×10^{-4}	9.18×10^{-6}	2.53×10^{-6}	2.33×10^{-4}	3.46×10^{-3}
**GWR9**	A19	1.89×10^{-4}	3.20×10^{-5}	3.03×10^{-2}	2.00×10^{-3}	4.50×10^{-3}	2.80×10^{-4}	1.33×10^{-4}	5.80×10^{-4}	2.75×10^{-1}	5.97×10^{-6}	1.01×10^{-6}	1.53×10^{-2}	3.16×10^{-4}	1.42×10^{-4}	8.86×10^{-6}	2.53×10^{-6}	5.24×10^{-5}	1.04×10^{-2}
	S20	9.43×10^{-3}	9.12×10^{-5}	1.91×10^{-2}	8.33×10^{-4}	4.14×10^{-3}	3.90×10^{-4}	3.07×10^{-4}	1.02×10^{-3}	2.39×10^{-1}	2.98×10^{-4}	2.89×10^{-6}	9.65×10^{-3}	1.32×10^{-4}	1.31×10^{-4}	1.23×10^{-5}	5.82×10^{-6}	9.19×10^{-5}	9.07×10^{-3}
	A20	9.14×10^{-4}	5.20×10^{-5}	1.72×10^{-2}	5.00×10^{-4}	6.56×10^{-3}	7.10×10^{-4}	7.60×10^{-4}	1.02×10^{-3}	3.25×10^{-2}	2.89×10^{-5}	1.65×10^{-6}	8.71×10^{-3}	7.91×10^{-5}	2.08×10^{-4}	2.25×10^{-5}	1.44×10^{-5}	9.19×10^{-5}	1.23×10^{-3}
	S21	4.65×10^{-3}	1.28×10^{-4}	2.93×10^{-3}	5.17×10^{-4}	2.68×10^{-3}	4.20×10^{-4}	1.12×10^{-4}	1.28×10^{-3}	2.63×10^{-1}	1.47×10^{-4}	4.05×10^{-6}	1.49×10^{-3}	8.17×10^{-5}	8.48×10^{-5}	1.33×10^{-5}	2.13×10^{-6}	1.15×10^{-4}	9.97×10^{-3}

Table S6. Hazard Index (HI) for the oral and dermal pathways and Total Hazard Index (THI) for those in the adult and children age categories exposed to spring waters collected in October 2019 (A19), May 2020 (S20), November 2020 (A20), and Spring 2021 (S21).

Spring	Season	Adult			Children		
		HI_{oral}	HI_{dermal}	THI	HI_{oral}	HI_{dermal}	THI
**GWR1**	A19	7.30×10^{-2}	4.92×10^{-3}	7.79×10^{-2}	9.28×10^{-2}	1.06×10^{-2}	1.03×10^{-1}
	S20	6.64×10^{-2}	5.18×10^{-3}	7.16×10^{-2}	8.46×10^{-2}	1.33×10^{-2}	9.78×10^{-2}
	A20	7.55×10^{-2}	7.23×10^{-3}	8.27×10^{-2}	9.61×10^{-2}	2.02×10^{-2}	1.16×10^{-1}
	S21	7.53×10^{-2}	5.90×10^{-3}	8.12×10^{-2}	9.58×10^{-2}	1.48×10^{-2}	1.11×10^{-1}
**GWR2**	A19	1.19×10^{-1}	5.79×10^{-3}	1.24×10^{-1}	1.51×10^{-1}	7.18×10^{-3}	1.58×10^{-1}
	S20	1.48×10^{-1}	7.27×10^{-3}	1.55×10^{-1}	1.88×10^{-1}	9.15×10^{-3}	1.97×10^{-1}
	A20	1.57×10^{-1}	8.03×10^{-3}	1.65×10^{-1}	2.00×10^{-1}	1.06×10^{-2}	2.10×10^{-1}
	S21	1.58×10^{-1}	1.23×10^{-2}	1.70×10^{-1}	2.01×10^{-1}	2.96×10^{-2}	2.30×10^{-1}
**GWR3**	A19	5.82×10^{-2}	3.13×10^{-3}	6.13×10^{-2}	7.41×10^{-2}	4.79×10^{-3}	7.89×10^{-2}
	S20	7.14×10^{-2}	3.54×10^{-3}	7.49×10^{-2}	9.09×10^{-2}	4.95×10^{-3}	9.58×10^{-2}
	A20	5.99×10^{-2}	3.23×10^{-3}	6.31×10^{-2}	7.62×10^{-2}	4.83×10^{-3}	8.11×10^{-2}
	S21	1.21×10^{-1}	1.27×10^{-2}	1.34×10^{-1}	1.54×10^{-1}	3.75×10^{-2}	1.91×10^{-1}
**GWR5**	A19	5.64×10^{-2}	2.83×10^{-3}	5.92×10^{-2}	7.18×10^{-2}	4.04×10^{-3}	7.58×10^{-2}
	S20	5.37×10^{-2}	2.71×10^{-3}	5.64×10^{-2}	6.84×10^{-2}	4.39×10^{-3}	7.28×10^{-2}
	A20	6.12×10^{-2}	3.68×10^{-3}	6.49×10^{-2}	7.79×10^{-2}	6.83×10^{-3}	8.48×10^{-2}
	S21	8.91×10^{-2}	9.38×10^{-3}	9.85×10^{-2}	1.13×10^{-1}	2.81×10^{-2}	1.42×10^{-1}
**GWR6**	A19	1.30×10^{-1}	6.42×10^{-3}	1.37×10^{-1}	1.66×10^{-1}	8.33×10^{-3}	1.74×10^{-1}
	S20	1.11×10^{-1}	5.27×10^{-3}	1.16×10^{-1}	1.41×10^{-1}	6.73×10^{-3}	1.48×10^{-1}
	A20	9.16×10^{-2}	5.19×10^{-3}	9.67×10^{-2}	1.17×10^{-1}	8.40×10^{-3}	1.25×10^{-1}
	S21	1.77×10^{-1}	1.71×10^{-2}	1.94×10^{-1}	2.26×10^{-1}	4.84×10^{-2}	2.74×10^{-1}
**GWR7**	A19	3.93×10^{-2}	3.27×10^{-3}	4.26×10^{-2}	5.00×10^{-2}	8.57×10^{-3}	5.86×10^{-2}
	S20	5.61×10^{-2}	5.79×10^{-3}	6.19×10^{-2}	7.13×10^{-2}	1.51×10^{-2}	8.65×10^{-2}
	A20	2.02×10^{-1}	1.22×10^{-2}	2.14×10^{-1}	2.57×10^{-1}	2.20×10^{-2}	2.79×10^{-1}
	S21	1.21×10^{-1}	1.47×10^{-2}	1.36×10^{-1}	1.54×10^{-1}	4.35×10^{-2}	1.98×10^{-1}
**GWR8**	A19	1.30×10^{-1}	6.26×10^{-3}	1.37×10^{-1}	1.66×10^{-1}	7.63×10^{-3}	1.73×10^{-1}
	S20	1.32×10^{-1}	6.52×10^{-3}	1.38×10^{-1}	1.68×10^{-1}	9.00×10^{-3}	1.77×10^{-1}
	A20	1.11×10^{-1}	5.81×10^{-3}	1.17×10^{-1}	1.41×10^{-1}	8.44×10^{-3}	1.50×10^{-1}
	S21	1.54×10^{-1}	1.58×10^{-2}	1.69×10^{-1}	1.96×10^{-1}	4.65×10^{-2}	2.42×10^{-1}
**GWR9**	A19	2.46×10^{-1}	1.48×10^{-2}	2.61×10^{-1}	3.13×10^{-1}	2.63×10^{-2}	3.39×10^{-1}
	S20	2.15×10^{-1}	1.19×10^{-2}	2.27×10^{-1}	2.74×10^{-1}	1.94×10^{-2}	2.93×10^{-1}
	A20	4.73×10^{-2}	3.91×10^{-3}	5.12×10^{-2}	6.02×10^{-2}	1.04×10^{-2}	7.06×10^{-2}
	S21	2.16×10^{-1}	1.04×10^{-2}	2.27×10^{-1}	2.75×10^{-1}	1.19×10^{-2}	2.87×10^{-1}
